# Signaling Metabolites in the Gut Microbiota–Organ Axes

**DOI:** 10.3390/cells15171558

**Published:** 2026-08-28

**Authors:** Diren Beyoğlu, Jeffrey R. Idle

**Affiliations:** College of Pharmacy and Health Sciences, Western New England University, Springfield, MA 01119, USA; diren.beyoglu@wne.edu (D.B.); jeff.idle@wne.edu (J.R.I.)

**Keywords:** gut microbiome, gut microbiota, gut–organ axes, SCFAs, polyamines, secondary bile acids, target organs, beneficial metabolites, deleterious metabolites

## Abstract

The gut microbiome is positioned at the center of a hub that contributes to both the physiology and pathology of multiple distant organs. It does this by releasing beneficial signaling molecules that are synthesized by various microbial species. Beneficial metabolites that cross from the gut lumen into the circulation include the short-chain fatty acids (SCFAs), a range of tryptophan metabolites (TRPMs), the major polyamines, and certain secondary bile acids. The SCFAs act on G-protein-coupled receptors (GPRs) and on histone deacetylases (HDACs) and, in so doing, suppress inflammation. Tryptophan metabolites act mainly through the nuclear receptor AhR, enhancing gut barrier and blood–brain barrier integrity. The polyamines stabilize nucleic acids and regulate cell proliferation. The secondary bile acids have varied effects on the liver, brain, and lung. In addition, the microbiome contributes to deleterious cometabolites, such as trimethylamine *N*-oxide, *p*-cresyl sulfate, and indoxyl sulfate. We predict that clinical laboratories in the future will assay patient samples for an amalgamation of beneficial and detrimental microbiota metabolites as biomarkers of both health and disease risk.

## 1. Introduction

The gut microbiota–organ axis refers to a complex, bidirectional, multi-channel communication network that allows microorganisms in the gastrointestinal tract to interact with distant organs throughout the body. Once viewed simply as a digestive tract, the gut is now understood to behave more like a primary homeostatic control center. It broadcasts and receives biochemical signals that influence everything from brain function to cardiovascular health [1,2]. For example, gut bacteria ferment dietary fiber to produce short-chain fatty acids (SCFAs), primarily, acetate, propionate, and butyrate, which enter systemic circulation to alter host cell metabolism and genetic expression in multiple organs, including the brain [3]. Moreover, the enteric nervous system acts as a “second brain” embedded in the gut wall. It relays immediate signals directly to the central nervous system via the long, bidirectional vagus nerve. This network is not only anatomical, but it also involves endocrine, humoral, metabolic, and immune routes of communication [4,5], as we have described [6,7,8].

In addition to the gut–brain axis, there are multiple other gut–organ axes. We have previously described the gut–liver axis [9] and the gut–lung axis [10]. The list also includes the gut–cardiovascular axis [11,12], the gut–kidney axis [13,14], the gut–skin axis [15,16,17], and the gut–bone axis [18,19]. Tripartite axes have also been described, such as the brain–gut–bone axis [20], the gut–kidney–heart axis [21], the brain–gut–kidney axis [22], the brain–gut–liver axis [23], the brain–gut–skin axis [24], and the brain–gut–adipose tissue axis [25,26]. It is now clear that multi-organ networks exist that involve four major components—the gut as the central hub, the brain via the nervous system, the liver via the portal vein, and the lungs and/or kidneys via circulatory immune pathways. For example, there is growing recognition that Parkinson’s disease is a systemic condition involving multiple organ–brain axes (lung–, liver–, heart–, muscle–, bone–, and gut–brain) in its pathogenesis [27].

In a recent review, Feng and colleagues, referring to the gut–organ axes, stated, “the communication mechanisms with distal organs have remained largely elusive” [28]. In a comprehensive review of the gut–organ axes, Lin et al. describe the bidirectional linkages between the gut microbiome and distal organs and pathologies that may be influenced by the microbiome [29], as we have recently described for the lung [10]. However, those authors [28,29] did not focus explicitly on the signaling molecules released by the gut microbiome, as we have done here. Here, we describe the production, release, transport routes, core biological activities, and extraintestinal organ effects of the principal beneficial and deleterious signaling metabolites of the gut microbiome.

## 2. The Hub: The Gut Microbiota

As we have previously stated [10], observation of living organisms in his own stool was first made by Antoni van Leeuwenhoek in 1681. He called these tiny motile organisms “animalcules” (small animals). This was perhaps both the discovery of bacteria and the first evidence of an intestinal microbiota [30]. The gut, in particular the colon, is the home to trillions of microorganisms, with a gradient of bacteria across the gastrointestinal tract from 10^1^ cells/g in the stomach, 10^3^ cells/g in the duodenum, 10^4^ cells/g in the jejunum, 10^7^ cells/g in the ileum, and 10^12^ cells/g in the colon [31]. Five phyla mainly comprise the bacteria of the human intestinal microbiota, although these proportions vary from study to study [9]. In one report [32], the proportions were Bacillota (formerly Firmicutes) (79-*Ruminococcus*, *Clostridium*, *Eubacteria*), Bacteroidota (formerly Bacterioidetes) (17-*Porphyromonas*, *Prevotella*), Actinomycetota (formerly Actinobacteria) (2.5-*Bifidobacterium*), Pseudomonadota (formerly Proteobacteria) (1%), and Verrucomicrobiota (0.1%). It has been estimated that the human gut microbiome consists of at least 1800 genera and ~15,000–36,000 species of bacteria [33]. Older estimates put the number of species at 500–1000 [34]. Bacteria are not the only microorganisms inhabiting the gut. Gut microbiota also comprises archaea, fungi, viruses and protozoa [35]. Viruses are the second most abundant microbe, mostly comprising phages, numbering 10^13^ to 10^15^ that may reduce the abundance of their target bacteria, such as *Bacteroides* and *Parabacteroides* spp. [36].

Regarding archaea, this is a group of single-celled microorganisms with characteristics distinct from bacteria, and that constitutes one of the three domains of life, along with bacteria and eukarya. They are generally considered nonpathogenic but this is based upon an incomplete understanding of these organisms [37]. Gut bacteria produce large amounts of hydrogen gas (H_2_) while breaking down dietary fibers. Archaea, such as *Methanobrevibacter smithii*, utilize this H_2_ as a fuel, combining it with CO_2_ to produce methane (CH_4_). This lowers the gas pressure within the gut (3 mol H_2_ + 1 mol CO_2_ = 4 mol→1 mol CH_4_) and permits the bacteria to continue to ferment dietary fiber. It was also reported that the Actinomycetota *Eggerthella lenta* oxidizes primary bile acids using a hydroxysteroid dehydrogenase that requires H_2_ levels to be low [38]. Finally, research has examined the complex molecular interactions between archaea and the human immune system [39]. Specific archaeal molecules, such as RNA and glycerolipids, stimulate immune responses via innate immune receptors such as Toll-like receptor 8 (TLR8) and C-type lectin domain family 4 member E (CLEC4E or MINCLE). In addition, metabolic byproducts of archaea, in particular methane, have demonstrated immunomodulatory effects through anti-inflammatory and anti-oxidative pathways [39].

Fungi constitute a small but highly influential portion of the human microbiota known as the “mycobiome.” While they represent less than 0.1% to 1% of the total microbial population in the gut, fungal cells are much larger than bacteria and contain highly complex genomes capable of executing thousands of unique metabolic and fermentative functions. Fungi inhabit the mucosal surface to maintain intestinal homeostasis and systemic immunity [40]. Functionally, the mycobiome is known to play a role in regulating innate and adaptive immune responses by cooperating with microorganisms and immune cells [41]. The fungal species comprising the intestinal mycobiome are largely yeasts, such as *Saccharomyces* (e.g., *S. cerevisiae*), *Candida* (e.g., *C. albicans*, *C. parapsilosis*), *Malassezia*, and *Cladosporium* [42]. A detailed listing of 16 fungal genera that occupy the mycobiome was given by Belvoncikova et al. [43]. The human mycobiome inhabits the oral cavity, gastrointestinal tract, respiratory tract, urogenital tract, and skin. Its composition can influence the gut–brain axis through immune and non-immune mediated crosstalk systems. Fungal–bacterial–host immune interactions are essential in preserving gut homeostasis, with fungi playing a role in facilitating immune responses such as Th17 cell activation [44].

Protozoa in the gut microbiota are not just random passengers; they function as active ecological engineers that shape the immune system, regulate bacterial populations, and maintain overall digestive health. While these single-celled organisms were historically viewed strictly as harmful parasites, advanced DNA sequencing has revealed that many are permanent, harmless commensals found in the gut of healthy individuals [45]. *Blastocystis hominis* and *Dientamoeba fragilis* are the commonest protozoa inhabiting the gut [46]. *Blastocystis* spp. predominantly phagocytose Enterobacteriaceae, including *E. coli*. By grazing on these fast-growing populations, *Blastocystis* prevents them from taking over the gut [47]. Blastocystis generally avoids or promotes the growth of beneficial, fiber-degrading bacteria like *Faecalibacterium prausnitzii* and members of the Ruminococcaceae family. This selective bacterial consumption is why persons with *Blastocystis* typically have higher overall gut bacterial diversity [48]. *Blastocystis* prevalence typically exceeds 5% in industrialized populations and is found generally in immunocompromised persons or those with close animal contact [49]. Protozoa interact with the host and immune system in three primary ways: (i) they produce metabolites like succinate, which act as signaling molecules to trigger the expansion of CD4^+^ T cells and drive localized tissue repair [50]; (ii) they stimulate host B cells to secrete high levels of IgA into the mucosa, which reinforces the gut barrier and provides long-lasting protection against pathogens [51]; and (iii) they can activate the host inflammasome to promote robust Th1 and Th17 adaptive immunity in the colon [50]. While bacteria dominate the microbiome landscape, microeukaryotic protists, once primarily viewed as mere parasites, are increasingly recognized as critical, dynamic regulators of mucosal immunity [51].

Specific groups of bacteria in the intestinal microbiota train immune cells to distinguish between harmless dietary antigens, beneficial commensals, and dangerous pathogens. They also help prevent autoimmune reactions by promoting immune tolerance [52]. Several groups of bacteria are responsible for this, including (i) segmented filamentous bacteria, specialized bacteria that strongly attach to colonic epithelial cells and are critical for inducing the development of Th17 cells, which protect against extracellular bacteria and fungi [53], recently assigned the name *Anisomitus miae* [54]; (ii) *Bacteroides* for example, *B. fragilis*, commensal bacteria that release specific molecules that antigen-presenting immune cells process to activate regulatory T cells (Tregs) and NKT cells, suppressing excess inflammation [55]; (iii) *Lactobacillus* spp. and *Bifidobacterium* spp., beneficial bacteria that promote local mucosal immune reactions by stimulating the production of IgA via B cells, acting as a barrier to neutralize pathogens and prevent overreactive inflammatory responses [56,57,58]; and (iv) certain *Klebsiella* spp., in particular, *K. aeromobilis* and *K. pneumoniae*, have been reported to induce strong Th1 cell responses in the gut, helping defend against intracellular pathogens [52]. These interactions between microbiota and immune cells operate on two levels. First, the bacteria release metabolites such as the SCFAs acetate and butyrate, which act directly on immune cells, enhancing epithelial barrier integrity and CD8^+^ T cell activity while also promoting immune tolerance. Other bacterial metabolites may also participate in these mechanisms, including, bile acids, tryptophan derivatives, and several emerging metabolites such as inosine, trimethylamine *N*-oxide (TMAO), and urolithin A [59]. Second, immune cells, such as dendritic cells, sample bacterial components and transport them to secondary lymphoid tissues, occasionally even to the thymus, to educate T cells [60].

Bacteriophages in the gut virome are critical in training the immune system. They do this by selectively culling bacterial populations to maintain balance, stimulating immune cell receptors, and acting as a physical shield along the intestinal mucus barrier [61,62,63,64]. Phages also interact directly with cellular pro-inflammatory cytokines, as well as reducing ROS overproduction [65,66,67]. Additionally, certain phages can cross the intestinal lining and enter the systemic circulation using a trojan horse mechanism [68]. Here, immune cells actively ingest these phages, activating virus-detecting receptors (like TLR9). This process recruits adaptive immune programs and causes the secretion of specific cytokines, training the immune system to distinguish between harmful and beneficial microbes [61]. Furthermore, the mycobiome and virome work in synergy with the bacterial microbiome to modulate host immunity [61]. The gut mycobiome and virome have recently been described as “neglected kingdoms,” which are gaining increased attention in relation to inflammatory bowel disease [69].

While archaea, fungi, and protozoa are not major producers of signaling metabolites, they certainly are important members of a eubiotic microbiome. Phages, on the other hand, may have profound effects upon microbial composition as described and therefore influence signal quality and strength of the microbiome hub.

In summary, the gut microbiota acts as an orchestra comprising bacteria, archaea, fungi, protozoa, and phages that engage in complex, dynamic crosstalk to train immune cells to recognize threats while maintaining tolerance to beneficial microorganisms, driving immune development through continuous biochemical and physical interactions.

## 3. Transmission of Metabolic Signals from the Hub

The microbiota hub acting as a command center transmits signals through the gut–organ axis using metabolites, immune cells, endocrine, humoral, and neuronal pathways.

### 3.1. Metabolic Signal Transmission

**Figure 1:**
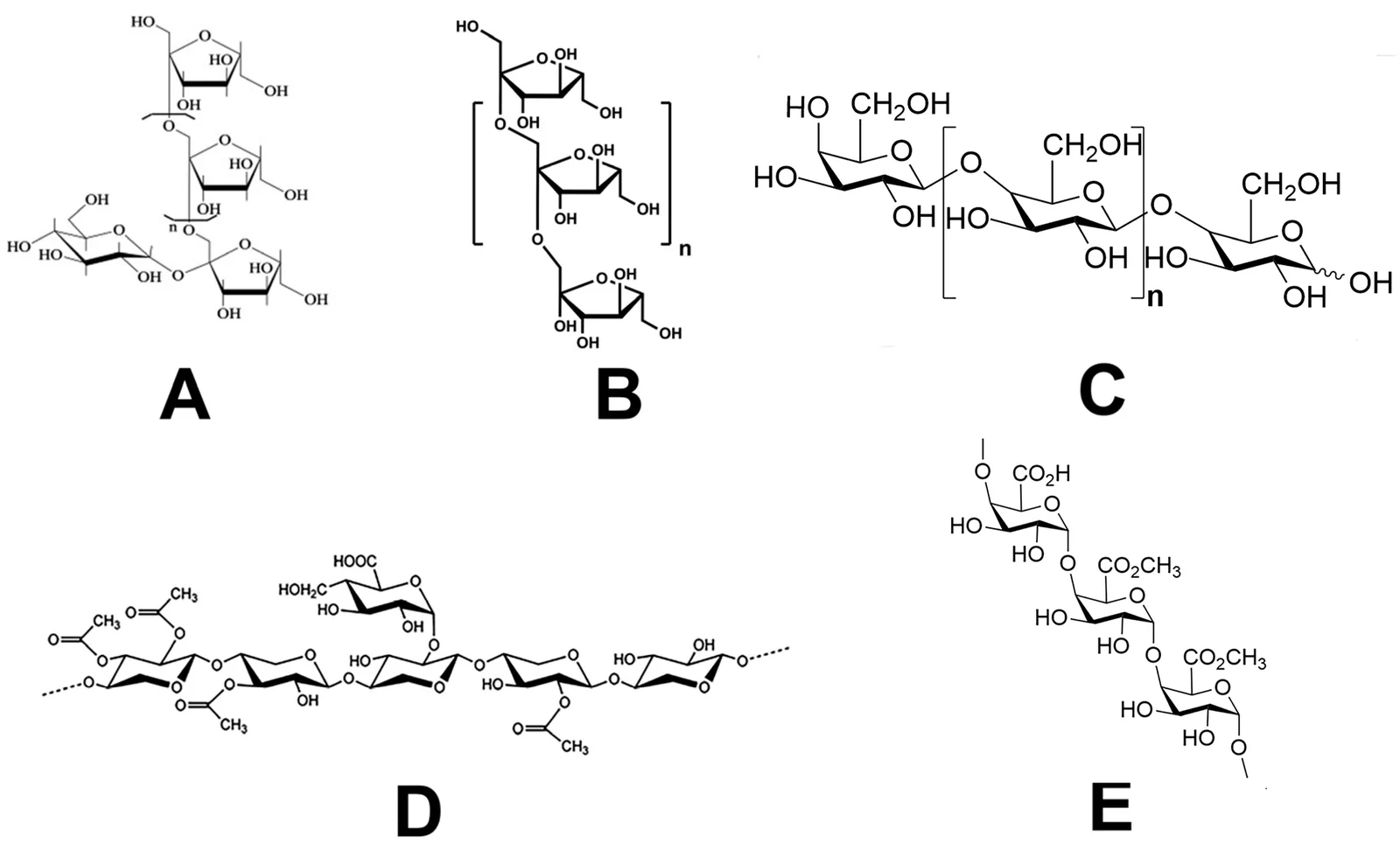
Chemical structures of the principal dietary fiber oligosaccharides. (**A**) **Inulin**, found in chicory root, artichokes, garlic, and onions; (**B**) **Fructooligosaccharides**, found in chicory root, artichokes, garlic, onions, leeks, asparagus, bananas, grapes [75,76], and blue agave (from which tequila is made); (**C**) **Galactooligosaccharides**, found in lentils, chickpeas, black beans, kidney beans, pinto beans, navy beans, split peas, cashews, pistachios, beetroot, broccoli, fennel, and butternut squash; (**D**) **Hemicellulose**, found in wheat, oats, barley, rice, peas, beans, lentils, potatoes, carrots, and sweet potatoes; (**E**) **Pectin**, found in apples, oranges, lemons, limes, grapefruits, passion fruit, quinces, plums, and carrots.

As mentioned above, the bacterial majority component of the gut microbiota synthesizes acetate and butyrate by the fermentation of soluble dietary fibers and resistant starches. The key fermentable fibers that cannot be digested by the host include inulin, a fructan and type of soluble dietary fiber and naturally occurring storage carbohydrate. Chemically, it is a polysaccharide made up of linear chains of fructose units connected by β (2→1) glycosidic bonds, with a single terminal glucose unit attached through a glycosidic α (1→2) linkage [70]. Oligosaccharides include fructooligosaccharides, comprising 2 to 60 fructose units [71] and galactooligosaccharides that are composed of 2 to 10 monosaccharide units, predominantly galactose, with a terminal glucose [72]. More complex dietary polysaccharides include pectin, a complex heteropolysaccharide with a chemical structure that is primarily a linear backbone of partially methylated and acetylated α-1,4-linked D-galacturonic acid units [73]. Finally, hemicellulose is a complex plant-derived polysaccharide made up of xylose (33.1–70.6%), glucose (6.7–17.2%) uronic acids (5.5–13.3%), arabinose (3.2–10.1%), galactose (4.8–6.8%), mannose (1.6–3.6%), and rhamnose (0–3.1%) [74]. The chemical structures of these dietary fibers are given in Figure 1.

On reaching the cecum, these dietary fibers undergo a fermentation process, mainly involving *Bacteroides*, *Bifidobacterium*, *Prevotella*, *Faecalibacterium*, *Roseburia*, *Butyrivibrio*, and *Ruminococcus* spp. [77]. The SCFAs generated include acetate (C2), propionate (C3), butyrate (C4), valerate (C5), caproate (C6), the last two found in smaller, trace amounts in the colon. The microbiota also produces the side-products L-lactate [78], CO_2_, H_2_ and CH_4_ [79] when it ferments dietary fiber to SCFAs. Both CO_2_ and H_2_ can be absorbed through the lumen wall into the bloodstream and exhaled by the lungs [80]. Additionally, these gases may be expelled as flatus or CO_2_ and H_2_ can be used by methanogenic archaea to produce methane. *Methanobrevibacter smithii* is the dominant methane-producing organism in the human colon, constituting up to 10% of all microbes in the colon of healthy human adults [81]. In contrast to dietary fiber, when the microbiota ferments unabsorbed dietary proteins and the branched-chain amino acids valine, leucine, and isoleucine, the branched-chain SCFAs isobutyrate, isovalerate, and 2-methylbutyrate are formed [79,82].

In addition to the high-fiber foods mentioned above, other functional foods contribute to the microbiome metabolic signaling to distant organs. For example, fermented foods, such as yogurt, kefir, kimchi, sauerkraut, and kombucha, deliver live beneficial microbes and organic acids, for example, lactate and acetate, broadening microbial diversity [83]. Moreover, polyphenol-rich foods, such as berries, green tea, dark chocolate, and extra virgin olive oil, act as substrates that specific gut microbes biotransform into smaller phenolic acids that inhibit pathogenic bacteria while encouraging the growth of protective strains, like *Akkermansia muciniphila* [84,85].

#### 3.1.1. Metabolic Signaling from the Gut Microbiota to the Gut Epithelium

**Figure 2:**
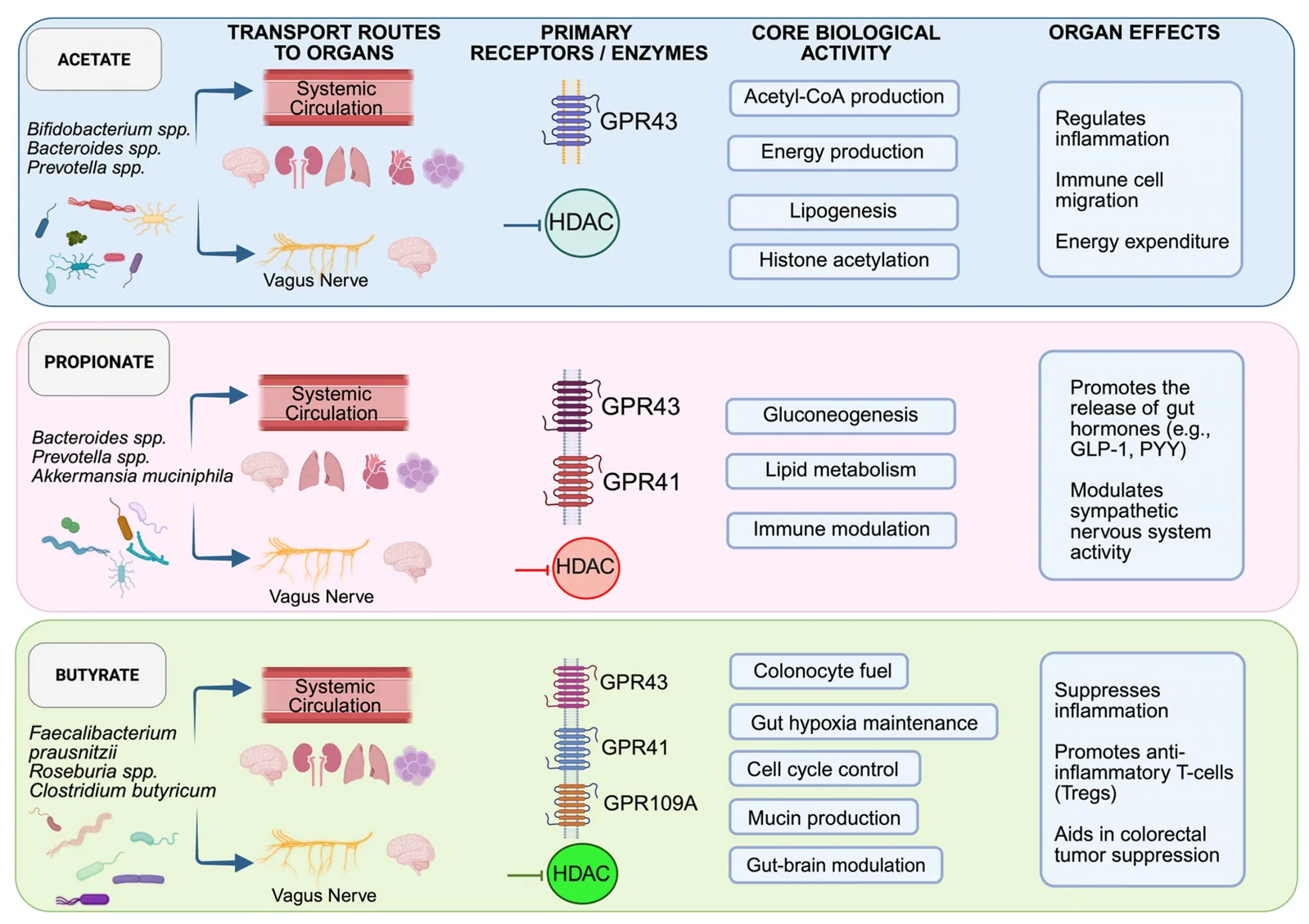
Microbial production and transport routes for acetate, propionate, and butyrate, their primary receptors (GPR41, GPR43, GPR109A) and inhibition of histone deacetylases (HDACs), leading to their core biological activities and specific organ effects.

Colonocytes rely on SCFAs for 60% to 70% of their total energy [86]. Butyrate is the principal fuel, undergoing β-oxidation that yields NADH and FADH_2_, which are used by the electron transport chain to generate ATP. Butyrate β-oxidation generates acetyl-CoA, which enters the TCA cycle. Each molecule of butyrate that undergoes β-oxidation, TCA cycle metabolism of the resulting acetyl-CoA, followed by oxidation of the energy carrier molecules NADH and FADH_2_ in the electron transport chain, generates 17 molecules of ATP [87]. Butyrate acts as a signaling molecule by binding to specific G-protein-coupled receptors on colonocytes, such as GPR41, GPR43, and GPR109A. This triggers the production of antimicrobial peptides and important gut hormones, like GLP-1 and PYY, which help regulate appetite and blood sugar [88]. SCFA signaling to colonocytes promotes the production of thick, protective mucus layers and strengthens “tight junctions” between cells, which prevents harmful bacteria and toxins from leaking into the bloodstream [88]. Inside the cells, especially butyrate and propionate act as histone deacetylase (HDAC) inhibitors. This alters gene expression to promote healthy cell turnover (proliferation and differentiation), prevent tumor formation, and reduce local inflammation [89]. By altering cytokine production and reducing luminal oxygen levels through their metabolism, SCFAs create a mildly acidic, oxygen-poor environment. This actively suppresses the growth of opportunistic pathogens while supporting beneficial, symbiotic bacteria [86]. The principal properties of SCFAs are given in Figure 2.

In inflammatory bowel disease (IBD), SCFAs act as crucial protective molecules that maintain intestinal homeostasis, repair the gut barrier, and suppress runaway inflammation [90]. As stated above, butyrate provides up to 70% of the daily energy requirements for colonocytes. In IBD, this energy supply is essential for cell regeneration and healing ulcerated mucosal tissues. Recent studies have illuminated a novel mechanism by which butyrate enhances mucosal defense through modulation of host-derived antimicrobial peptides. Butyrate was shown to inhibit histone deacetylases in epithelial and immune cells, leading to upregulation of multiple host defense peptides. This epigenetic regulation strengthened bacterial clearance and reduced inflammatory markers in experimental models, demonstrating a direct link between SCFA-mediated chromatin remodeling and intestinal resilience [91,92].

#### 3.1.2. Metabolic Signaling from the Gut Microbiota to the Brain

The SCFAs produced by the gut microbiota have major effects on the brain through the gut–brain axis. Acetate, propionate, and butyrate enter the brain primarily by crossing the blood–brain barrier (BBB) using the monocarboxylate transporter MCT1 (SLC16A1) located on brain microvascular endothelial cells, as all these SCFAs have been observed in CSF at higher concentrations than in blood [93]. However, administration of sodium butyrate to mice by gavage elicited reduced food intake, while in mice that had undergone subdiaphragmatic vagotomy, this effect was abolished [94]. These investigations in rodents demonstrate that butyrate communicates with the brain via the vagus. It has been reported that it does so by GPR41/43 receptor signaling [95]. Individual SCFAs have a wide range of effects on brain physiology. Acetate has a number of effects on the brain, including as an energy source for both astrocytes and neurons [96,97]. Although glucose is the major obligatory fuel for all brain cells, acetate provides important amounts of energy to functioning astrocytes [98]. In particular, acetate was reported to be metabolized in GABAergic neurons, as a major contributor to GABA synthesis [99]. Acetate also enhanced neuronal plasticity and improved survival after ischemic brain injury [100]. Acetate furthermore restored metabolic function and cognitive ability after chronic sleep disruption in a mouse model [101].

Propionate, in contrast, protects the cerebrovascular endothelium from oxidative stress and helps maintain normal physiological function of the blood–brain barrier via specific signaling [102]. Animal models of type-1 diabetes suggest propionate can suppress oxidative stress, stimulate cerebral microcirculation, and reduce neuron loss in the hippocampus [103]. Microglia, the innate immune cell of the brain, plays a major rolein Alzheimer’s Disease (AD). Microglia attempt to clear and degrade amyloid beta (Aβ), the protein aggregates that drive AD pathogenesis, by using phagocytic machinery, prompting damaging neuroinflammation in the process. Propionate blunted the early inflammatory response driven by Aβ, downregulating the expression of many Aβ-stimulated immune genes [104]. However, elevated propionate concentrations can exhibit neurotoxic properties, for example, in propionic acidemia, a rare metabolic disorder caused by mutations in the propionyl-CoA carboxylase gene, leading to major neurological dysfunction [105]. Furthermore, depending on concentration, propionate may have antidepressant or so-called prodepressant effects [106].

Butyrate has been said to improve brain health through a number of pathways, including inhibition of HDAC [107,108,109], binding to certain GPCRs [108,109,110], and as an energy metabolite [108,109]. These diverse modes of action make butyrate well suited for unraveling the wide range of metabolic distortions that are frequently encountered in neurological disorders [111,112,113,114].

The enteric nervous system (ENS), containing over 100 million nerve cells, is lined with microbial receptors. Gut microbes directly stimulate afferent neurons, sending rapid electrical signals to the brain via the vagus nerve, the body’s central information highway [115,116]. Approximately 80% to 90% of the fibers in the vagus nerve are afferent (sensory), meaning they transmit signals from internal organs and viscera to the brainstem. Early anatomists ultimately assigned the Latin name *vagus*, which literally translates to “the wanderer.” This perfectly captured how the nerve winds from the brainstem down into the chest and abdomen to interact with nearly every major organ, a characteristic first described by the Roman physician Galen of Pergamum (129–199 CE) [117]. Galen, whose work would dominate European medicine for 1500 years, recognized the importance of the connection between the brain and visceral organs [118].

The microbiota synthesizes neurotransmitters identical to those produced by the host, including serotonin (5-HT), dopamine, and GABA [119]. The gut is responsible for producing 90 to 95% of the body’s 5-HT [120]. It is generally believed that 5-HT, dopamine, and GABA produced in the gut and that enter the circulation do not cross the blood–brain barrier (BBB) [121,122,123]. Gut-derived 5-HT produced by enterochromaffin (EC) cells regulates bowel movements and intestinal inflammation. EC cells also signal to the central nervous system indirectly by releasing 5-HT that acts on 5-HT_3_ receptors on vagal efferent fibers in a paracrine fashion [124], stimulating the vagus nerve and sending impulses to the nucleus tractus solarius in the brainstem, influencing memory [125], mood, and stress response [126]. 5-HT that enters the circulation is taken up and concentrated in platelets [127]. EC cells increase 5-HT synthesis by upregulation of tryptophan hydroxylase (TPH1) in response to the SCFAs acetate and butyrate [128,129] produced by *Bacteroides*, *Prevotella*, and *Bifidobacterium* spp., various *Streptococcus*, *Clostridium*, and *Ruminococcus* spp., together with *Akkermansia muciniphila* [130]. In addition, tryptophan metabolites, such as tryptamine that is produced by the decarboxylation of tryptophan by *Clostridium sporogenes* and *Ruminococcus gnavus* [129,131], also stimulates EC cells to produce 5-HT via 5-HT_4_ receptors [129]. Furthermore, tryptophan is also biotransformed by *Edwardsiella tarda* into various indole metabolites that activate TRPA1 receptors on EC cells to stimulate 5-HT production.

Primary bile acids produced in the liver from cholesterol and released into the bile as glycine and taurine conjugates are converted primarily by 7α-dehydroxylation by the microbiota into secondary bile acids. Members of the phylum Bacillota, principally *Clostridium scindens* yield deoxycholic acid and lithocholic acid, after the primary bile salts are first deconjugated (glycine and taurine removed by bile salt hydrolase [EC 3.5.1.24]) by *Lactobacillus*, *Bifidobacterium*, *Bacteroides*, *Clostridium*, *Enterococcus* spp. [132]. Secondary bile acids bind to the TGR5 receptor (Takeda G-protein-coupled receptor 5) that is highly expressed on colonic EC cells causing the release of 5-HT that both stimulates afferent vagal fibers via 5-HT_3_ receptors, as stated above, and increases gut motility by the release of acetylcholine and CGRP via 5-HT_4_ receptors [133]. Like SCFAs, secondary bile acids transmit signals through afferent vagal fibers to the brainstem [134]. Signals sent from the gut to the brainstem by secondary bile acids serve several critical purposes in the gut–brain axis. This enhances gut satiety signals (like CCK), signaling the brainstem and hypothalamus to stop eating [135].

Signaling via the gut–brain axis results in the continuous central regulation of essential survival, metabolic, and emotional functions. In the brain, these incoming signals influence mood, stress responses, food cravings, and the perception of visceral pain, forming a vital bridge between the digestive tract and overall mental health. Of particular neurological and psychiatric relevance, the gut–brain axis influences neurodevelopment, neurotransmission, and behavior [136]. As stated above, the microbiota produce SCFAs and tryptophan metabolites that act on EC cells to release 5-HT that stimulates action potentials in the vagus, which are sensed in the nucleus tractus solarius of the brainstem. The brain then integrates these signals and can modulate serotonergic neurons in the dorsal raphe nucleus as well as the noradrenergic neurons in the locus coeruleus. This communication impacts emotional regulation, stress responses, and immune modulation [137]. Because the gut–brain axis affects mood, behavior, and stress levels, disruption of these afferent signals by, for example, dysbiosis resulting in a leaky gut, can lead to conditions like anxiety, depression, and cognitive fatigue [136,138,139,140].

Importantly, the global prevalence of major depressive illness and anxiety disorders is estimated to be 3.1% and 4.8%, respectively [141]. Several specific strategies are being actively researched to target the gut microbiota and the gut–brain axis to leverage novel treatment modalities for these conditions. One of these involves the use of probiotics, prebiotics, and synbiotics [142], which, in this specific clinical context have been termed “psychobiotics,” defined as “a live organism that, when ingested in adequate amounts, produces a health benefit in patients suffering from psychiatric illness” [143]. Psychobiotics used include *Bifidobacterium longum* (strains 1714 and R0175). These are some of the most extensively studied psychobiotics in clinical trials. They are shown to reduce cortisol levels, alleviate symptoms of anxiety, and improve cognitive performance. In addition, *Bifidobacterium breve*, known to reduce anxiety behaviors and, in combination with other strains, shows promise in slowing cognitive decline. Finally, *Bifidobacterium infantis*, often studied for its anti-inflammatory effects, has been linked to positive benefits in patients with chronic fatigue syndrome and depression [144]. In the *Lactobacillus* group, *Lacticaseibacillus rhamnosus* (Strain JB-1) is one of the most notable psychobiotics, proven in landmark animal and human investigations to reduce anxiety and stress hormones by altering brain GABA receptor expression. Also, *Lactiplantibacillus plantarum* (e.g., Strain JYLP-326) has been found to boost mood-regulating neurotransmitters (5-HT and dopamine) and was effective in alleviating severe stress and exam anxiety [145]. Moreover, *Lactobacillus helveticus* (strain R0052), frequently used alongside *B. longum* reduces symptoms of depression, anxiety, and psychological distress. Finally, *Limosilactobacillus reuteri* has been demonstrated in a mouse model to increase GABA and promote anti-inflammatory effects through the vagus nerve [146,147]. Other notable bacteria evaluated as psychobiotics include *Akkermansia muciniphila*, *Lacticaseibacillus casei*, and *Lactococcus lactis* [148]. Nevertheless, it must be declared that the findings with psychobiotics are based upon preliminary clinical evidence and that large-scale randomized clinical trials will be required to substantiate or refute the validity of the current claims that psychobiotics may be useful in the treatment of depression, for example.

One of the most important tasks carried out by the gut–brain axis is signaling to the brain that fuel is required by the gut. This occurs through three primary pathways. The first is hormone signaling. Specialized neuroendocrine cells lining the gastrointestinal tract constantly monitor the digestive environment. When devoid of food, the production of ghrelin is increased, mainly by the stomach, signaling the brain to initiate foraging and eating behaviors. Ghrelin is a 28 amino acid, appetite-stimulating peptide hormone that requires activation by octanoylation of the serine-3 [149] by an enzyme known as ghrelin *O*-acyltransferase (GOAT) [150]. Ghrelin in the circulation, both in its octanoylated and free forms, crosses the BBB and is concentrated in the olfactory bulb [151]. Ghrelin binds to the growth hormone secretagogue receptor (GHSR-1a), which was also believed to actively transport ghrelin across the BBB but experiments in *Ghsr* null mice established that this was not the case [151]. There has therefore been much speculation about how ghrelin enters the brain to gain access to the arcuate nucleus neurons (ARC neurons) at the base of the hypothalamus. One proposal has been access through a specialized group of CNS structures called the sensory circumventricular organs, which are not protected by the blood–brain barrier [152]. Ghrelin also activates GHSRs located directly on the vagus nerve in the gut [153]. The vagus then relays these hunger signals straight to the nucleus tractus solitarius in the brainstem, which then communicates them to the hypothalamus [154].

Many neurotransmitters found in the brain can be produced by the members of the gut microbiota. These include dopamine, norepinephrine, 5-HT, GABA, acetylcholine, and histamine [155], together with glutamate, tyramine, phenylethylamine, and tryptamine [121]. The individual bacteria that produce each neurotransmitter have been tabulated [121,155]. Neurotransmitters such as dopamine, 5-HT, and GABA can pass through the intestinal wall and enter the bloodstream [121]. However, the BBB is impermeable to these neurotransmitters. In fact, specific transporters are responsible for efflux of GABA across the BBB [156,157]. The brain capillary endothelial cells (BCECs) of the BBB contain specialized efflux transport proteins of the ATP-binding cassette (ABC) transporter family, such as ABCB1 (P-glycoprotein, Pgp) and ABCG2 (breast cancer resistance protein, BRCP), which transport numerous precursors and metabolites of neurotransmitters [158]. BCECs also express 5-HT and norepinephrine efflux transporters [159]. Overall, the hypothesis was developed that the BBB acts as a CNS detoxifying system for both endogenous substrates and xenobiotics in the brain [160].

Therefore, the release of neurotransmitters from the gut lumen into the circulation does not constitute a component of the gut–brain axis.

#### 3.1.3. Metabolite Signaling from the Gut Microbiota to the Lung

We have recently reported on the crosstalk between the gut and lung microbiomes, known as the gut–lung axis [10]. Instead of bacteria physically migrating in large numbers, the gut microbiome communicates with the lungs using bioactive metabolites and immune cells that travel via the bloodstream and lymphatic system. These authors [161] asked the question, “how might alterations in the gut microbiome influence respiratory health through the gut–lung axis?” As we reported previously, the gut–lung axis plays a role in several principal pulmonary diseases, including asthma, chronic obstructive pulmonary disease, cystic fibrosis, bronchogenic carcinoma, COVID-19, interstitial lung diseases, pneumonia, and tuberculosis [10]. The three principal groups of microbial metabolites that pass through the gut–lung axis are SCFAs, tryptophan metabolites (TRPMs), and polyamines. As stated above, the SCFAs acetate, propionate, and butyrate are produced from fermentation of dietary fiber by the anaerobic commensals *Bacteroides*, *Bifidobacterium*, *Prevotella*, *Faecalibacterium*, *Roseburia*, *Butyrivibrio*, and *Ruminococcus* spp. [77,162]. The SCFAs then cross the gut epithelial barrier by both passive diffusion and active transport by the transporter SLC5A8. On reaching the bone marrow, SCFAs reprogram immune progenitor cells, making dendritic cells and macrophages less reactive to harmless allergens, with implications for pulmonary pathophysiology [10]. In the lung, SCFAs bind GPR41 and GPR43 on epithelial cells, suppressing allergic airway inflammation and reducing tissue damage [163]. Further details of the core biological activities, together with the key local and systemic effects of acetate, propionate, and butyrate are given in Figure 2. In the lung, SCFAs act as powerful modulators of pulmonary immunity, barrier function, and inflammation.

**Figure 3:**
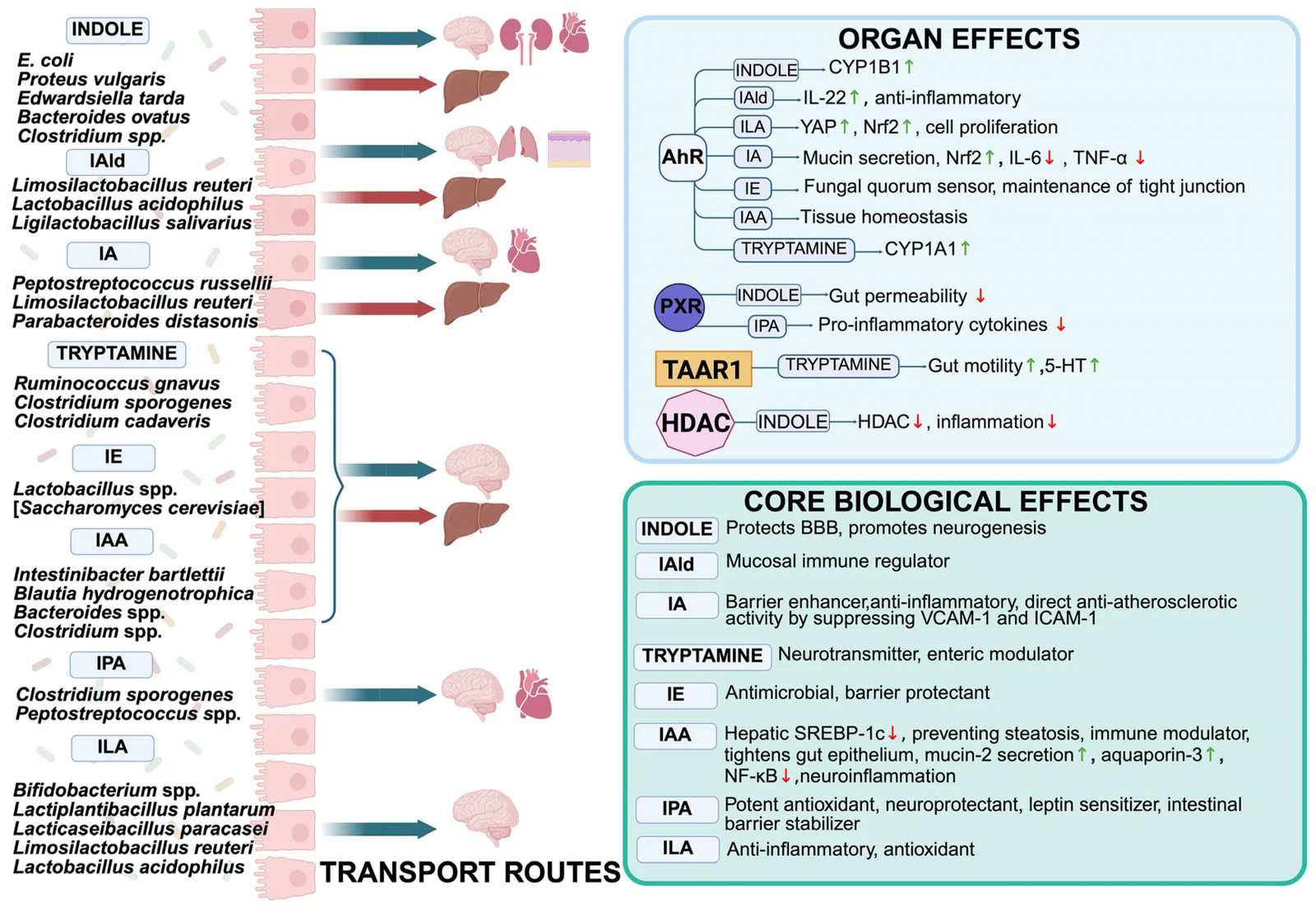
Microbial production of eight tryptophan metabolites, their core biological effects, and their transport routes through the gut–organ axes. Blue arrows signify systemic circulation; red arrows signify the hepatic portal vein. For the abbreviations of the TRPMs, see text above. Multiple organ effects of TRPMs are mediated by the nuclear receptors AhR and PXR. Tryptamine also acts on the trace amine-associated receptor 1 (TAAR1). Indole acts on both PXR to reduce gut permeability and on AhR to block histone deacetylases (HDAC), which results in attenuated inflammation. Green up arrow represent increased; red down arrow represent decreased.

The TRPMs are produced by a number of commensal bacteria, including *Lactobacillus* spp., and include indole, tryptamine, indole-3-ethanol (IE; tryptophol), indole-3-propionic acid (IPA), indole-3-lactic acid (ILA), indole-3-acetic acid (IAA), skatole (3-methylindole), indole-3-aldehyde (IAld) and indole-3-acrylic acid (IA) [164]. The primary receptors, core biological activities, and key systemic and local effects of these metabolites are shown in Figure 3. It is clear that certain indoles have effects that are relevant to multiple organs.

**Figure 4:**
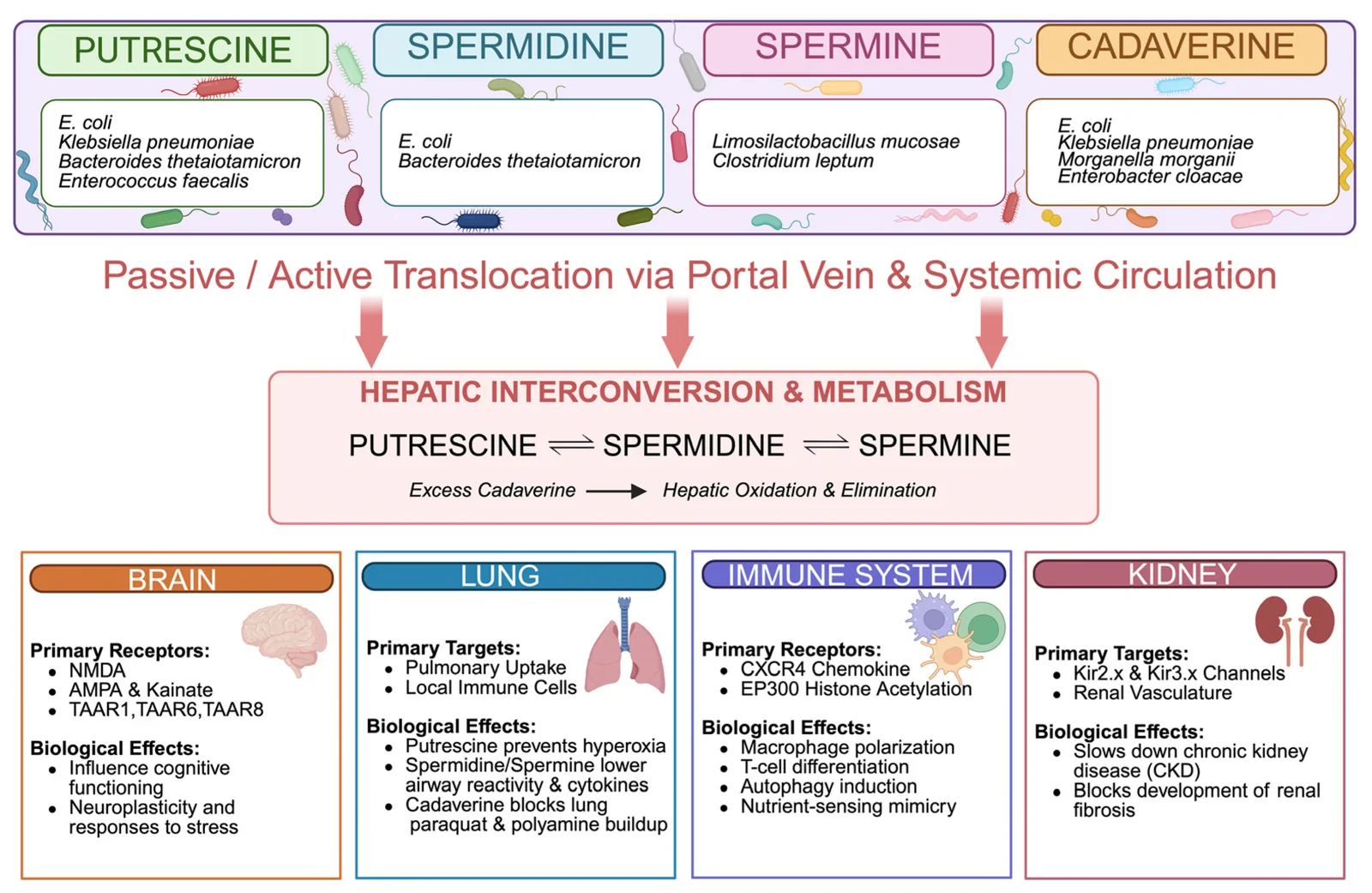
Microbial production of polyamine metabolites, their transport routes through the gut–organ axes, their primary receptors or targets, and their principal biological effects on target organs.

As stated above, the third group of gut microbial metabolites that enter the gut–lung axis are the polyamines. The polyamines (putrescine, spermidine, and spermine) are involved in cellular growth, proliferation, and differentiation. Unlike SCFAs, which act through dedicated G-protein-coupled receptors, microbial polyamines do not target a specific, dedicated polyamine receptor. Instead, they function as polycationic molecules. Their highly positive charge allows them to diffuse across membranes or enter cells via an active polyamine transport system. Once inside or interacting with cell membranes, they modulate cellular behavior by binding directly to a variety of established intracellular structures and surface ion channels. The biological mechanisms and clinical significances are given in Figure 4.

**Figure 5:**
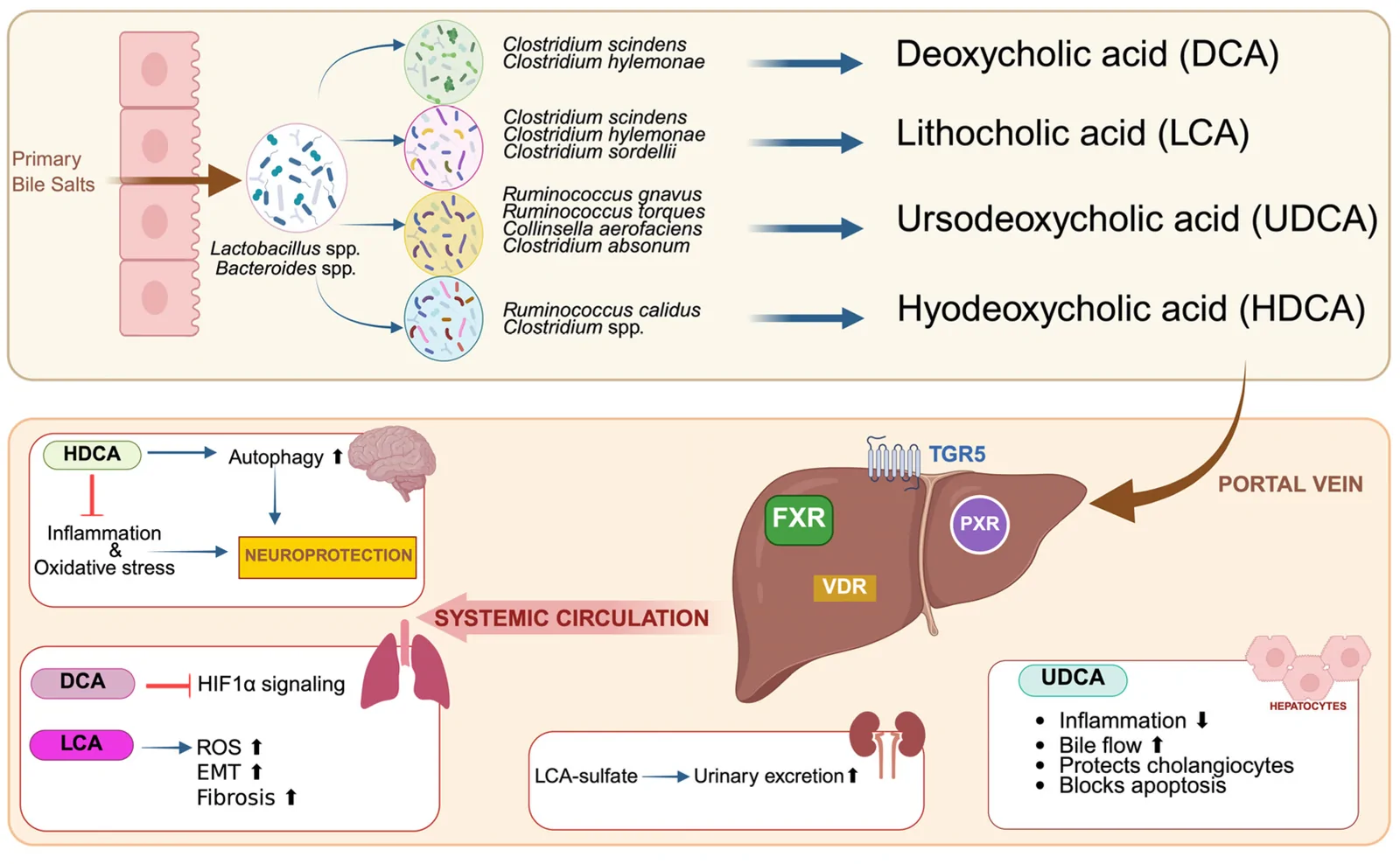
The production and transport through the gut–organ axes of the secondary bile acids.

The production and transport through the gut–organ axes of the secondary bile acids is given in Figure 5. The microbial formation of the secondary bile acids DCA, LCA, UDCA, and HDCA from primary bile salts in the gut lumen, transport to the liver in the portal vein where they act on nuclear receptors FXR, PXR, the vitamin D receptor (VDR), and the Takeda G-protein-coupled receptor 5 (TGR5). Secondary bile acids in the liver regulate bile acid synthesis from cholesterol (FXR), regulate bile flow and reduce hepatitis (TGR5), increase metabolism and detoxication of xenobiotics (PXR), regulate lipid metabolism in hepatocytes, blocks fibrosis by hepatic stellate cells, and reduce tissue inflammation, oxidative stress, and insulin resistance in hepatic Kupffer cells (VDR). The secondary bile acid LCA is highly hydrophobic and to facilitate its elimination it is conjugated with sulfate and excreted through the kidney. In hepatocytes, UDCA reduces inflammation, increases bile flow, protects cholangiocytes, and blocks apoptosis. Secondary bile acids enter the hepatic vein and the general circulation for transport to the target organs brain (HDCA blocks oxidative stress and inflammation) and lung (DCA blocks HIF1α signaling and stimulates autophagy; while LCA increases oxidative stress, epithelial–mesenchymal transition (EMT), and fibrosis).

Figure 2, Figure 3, Figure 4 and Figure 5 demonstrate that multiple gut microbiota metabolites, in particular SCFAs, tryptophan metabolites, polyamines, and secondary bile acids gain access to the gut–organ axes. Therefore, at least 20 gut microbial-produced metabolites act as signaling molecules that alter the physiology of target organs.

As we have stated elsewhere [10], the gut–lung axis significantly influences various respiratory conditions. Gut microbiota dysbiosis directly affects immune responses, altering disease severity in asthma, chronic obstructive pulmonary disease (COPD), cystic fibrosis (CF), and lung infections such as pneumonia and COVID-19. Gut microbiota metabolites, particularly SCFAs, tryptophan catabolites, and secondary bile acids, regulate pulmonary immunity through G-protein-coupled receptors, histone deacetylase inhibition, and aryl hydrocarbon receptor signaling [10].

#### 3.1.4. Metabolite Signaling from the Gut Microbiota to the Liver

The gut microbiome communicates directly with the liver via the gut–liver axis, an anatomical and biochemical highway. Because the venous blood from the gut drains directly into the liver through the portal vein, the liver is constantly bathed in gut-derived signals, maintaining a delicate balance between immunity and metabolism [9,165]. As stated above, gut bacteria break down dietary fibers to create signaling molecules. In particular, SCFAs like butyrate, propionate, and acetate travel to the liver, where they activate hepatic AMPK (AMP-activated protein kinase). This inhibits lipogenesis and reduces gluconeogenesis, which helps prevent conditions like metabolic dysfunction-associated steatotic liver disease (MASLD) [166,167]. As stated above, SCFAs act as HDAC inhibitors, directly altering chromatin structure to upregulate beneficial genes [168,169], such as those responsible for fatty acid oxidation and ketogenesis [170,171].

Conversely, bacteria can produce deleterious metabolites by converting choline (mostly from dietary meat and eggs [172]) into trimethylamine (TMA), which is further metabolized in the liver to its *N*-oxide TMAO, principally by the flavin-containing monooxygenase FMO3 [173]. TMAO triggers liver inflammation and fat accumulation by three separate mechanisms. First, TMAO shifts the composition of the hepatic bile acid pool toward bile acids that act as antagonists to the Farnesoid X Receptor (FXR) by inhibiting key enzymes for bile acid synthesis (such as CYP7A1 and CYP27A1). When FXR signaling is suppressed, the liver increases bile acid synthesis and upregulates de novo lipogenesis, directly enhancing the accumulation of hepatic triglycerides and lipid droplets [174,175]. Second, TMAO has been identified as an agonist that selectively triggers the PERK-mediated pathway of ER stress response. The chronic overactivation of this pathway disrupts protein folding homeostasis and directly drives lipid droplet accumulation. Chronic activation of these ER stress pathways also triggers downstream NLRP3 inflammasome signaling, increasing the production of ROS and pro-inflammatory cytokines like IL-1β leading to hepatic inflammation, and fibrosis [176]. Third, by the production of ROS, TMAO accelerates mitochondrial dysfunction, through interaction with HSP90β [177,178]. This cellular damage impairs oxidative phosphorylation, disrupts fatty acid metabolism, and boosts the release of pro-inflammatory cytokines that exacerbate hepatocyte injury [179].

As well as the beneficial metabolites acetate, propionate, and butyrate, and the detrimental TMA produced by the gut microbiota that reach the liver through the gut–liver axis, several additional metabolites are worthy of mention. For example, several of the TRPMs given in Figure 3 are known to reach the liver through the gut–liver axis, in particular, indole, indole-3-acetate (IAA), indole-3-propionate (IPA), tryptamine, and indole-3-aldehyde (IAld). In the liver, these metabolites exert protective and metabolic effects primarily by acting as ligands for the nuclear receptors, AhR and PXR [180,181].

In addition to the TRPMs, the aromatic amino acids phenylalanine and tyrosine are converted to phenolic metabolites such as *p*-cresol (4-methylphenol) by the 13 families Bacteroidaceae, Bifidobacteriaceae, Clostridiaceae, Enterobacteriaceae, Enterococcaceae, Eubacteriaceae, Fusobacteriaceae, Lachnospiraceae, Lactobacillaceae, Porphyromonadaceae, Staphylococcaceae, Ruminococcaceae, and Veillonellaceae [182]. Bacillota is therefore the dominant phylum synthesizing *p*-cresol. In vitro experiments appeared to show that four microbiota species were the major producers of *p*-cresol from phenylalanine and tyrosine—*Blautia hydrogenotrophica*, *Clostridioides difficile*, *Olsenella ulli*, and *Romboutsia liuseburensis* [183]. The complex metabolic conversion of the 6–18 g of proteins and peptides that enter the large intestine every day into *p*-cresol, then *p*-cresyl sulfate (PCS) and *p*-cresyl glucuronide in the liver has been mapped out [182]. Once formed in the liver, PCS acts as a harmful uremic toxin rather than a beneficial metabolite. It induces oxidative stress, depletes glutathione, and contributes to insulin resistance by promoting lipid accumulation in liver tissue. It is significantly less toxic than its free, unconjugated form, but still causes cellular and metabolic damage by enhancing oxidative stress [184]. Moreover, PCS induces the production of ROS in endothelial cells. This triggers endothelial dysfunction, arterial stiffness, and vascular calcification, significantly increasing the risk of heart failure and cardiovascular mortality [185]. Furthermore, accumulation of PCS accelerates the progression of chronic kidney disease (CKD) by inducing renal fibrosis and tubular cell damage. Because PCS is strongly protein-bound, it is poorly cleared by standard hemodialysis protocols [186,187]. Additionally, PCS alters bone metabolism by inducing osteoblast dysfunction, leading to decreased bone quality and strength [188]. PCS also crosses the blood–brain barrier and has been shown to induce neuroinflammation in a mouse model and apoptosis in neuronal cells. High levels are correlated with neurodevelopmental abnormalities and conditions such as depression and anxiety [189]. Interestingly, 17 studies conducted in Italy, France, Georgia, Slovenia, Latvia, and China reported that the concentration of *p*-cresol and PCS was higher in the urine of children with autism spectrum disorder vs. controls. Three animal studies have demonstrated that *p*-cresol caused autism-related symptoms in mice, and that mice could be restored by a fecal microbiota transplant from healthy mice [190].

As we have stated elsewhere [9], a panoply of metabolites, both beneficial and disadvantageous, are produced by the microbiota, including ethanol, secondary bile acids, trimethylamine, indole, and the quorum sensors acyl homoserine lactones that may contribute to hepatocellular carcinoma but this has yet to be fully investigated.

#### 3.1.5. Metabolite Signaling from the Gut Microbiota to the Kidney

Both beneficial and harmful metabolites pass through the gut–kidney axis. As with the brain, lung and liver, the beneficial SCFAs produced by the gut microbiota also pass through the gut–kidney axis. In the kidneys, they act as critical signaling molecules that reduce inflammation, suppress renal fibrosis, improve mitochondrial function, modulate energy metabolism, and help regulate blood pressure [191,192,193,194,195]. In addition to acetate, propionate, and butyrate (Figure 2), secondary bile acids have beneficial effects on the kidney. LCA and UDCA (Figure 5) act as signaling molecules in the kidneys, regulating fluid balance and protecting against inflammation [196]. By activating receptors such as FXR and TGR5, they control urine concentration [197], reduce kidney fibrosis [198], and suppress oxidative stress by activating FXR and TGR5 that downregulate ROS production and enhance mitochondrial defense systems [199].

The TRPMs, especially IPA and IAA (Figure 3), serve as natural ligands for the AhR. When these metabolites reach the kidneys, they activate AhR pathways, which suppress inflammation, reduce oxidative stress, and help maintain the integrity of renal cells [200]. In opposition to these beneficial effects of IPA and IAA, under conditions of gut dysbiosis, tryptophan metabolism shifts to produce high levels of uremic toxins, most notably indoxyl sulfate. Initially, indole is formed by the gut microbiota from tryptophan by microbes possessing tryptophanase. These include *Escherichia coli*, *Proteus vulgaris*, *Klebsiella oxytoca*, and *Bacteroides thetaiotaomicron* [201]. Once indole diffuses across the gut wall and enters the hepatic portal vein, it is further metabolized in two steps in the liver, first predominantly by CYP2E1-mediated 3-hydroxylation to indoxyl [202] and secondly and selectively by SULT1A1 [203]. When indoxyl sulfate (IS) reaches the kidneys, it promotes cellular damage and oxidative stress [204], inflammation [205,206], fibrosis [207], pro-inflammatory (M1) polarization of macrophages [208], and accelerates the progression of CKD [209]. Like IS, *p*-cresyl sulfate (PCS), derived from phenylalanine and tyrosine by the microbiota, is also a uremic toxin, which promotes inflammation, endothelial dysfunction, and fibrosis [210]. It should be stated that IS/PCS accumulation is primarily due to reduced renal clearance rather than increased colonic production in patients with CKD.

Microbially derived polyamines (Figure 4) are readily absorbed by the gut, enter systemic circulation, and travel to the kidneys. They profoundly impact kidney physiology in a dose-dependent manner by driving cellular growth [211], regulating mitochondrial homeostasis [212], modulating intracellular calcium levels [213], and mitigating inflammation and fibrosis [214]. The bacteria that contribute to polyamine biosynthesis in the gut that then pass through the gut–kidney axis are *Lactiplantibacillus plantarum*, *Lacticaseibacillus casei*, *Bifidobacterium longum*, *Bifidobacterium adolescentis* (cross-feeding with *Escherichia coli* and *Enterococcus faecalis*), *Enterococcus faecium*, *Bacillus subtilis*, *Streptococcus thermophilus*, and *Proteus* spp. [215,216,217,218,219,220,221].

#### 3.1.6. Metabolite Signaling from the Gut Microbiota to the Skin

The integumentary system is the body’s outermost layer, encompassing the skin, hair, nails, and associated glands. As the body’s largest organ, its primary function is to act as a physical barrier that protects internal tissues from pathogens, sunlight, injury, and dehydration [222]. The bidirectional gut–skin axis directly influences skin inflammation, barrier function, and overall complexion [16,223,224]. The gut–skin axis plays an important role in psoriasis [225], atopic dermatitis [226,227], rosacea [228], acne [229], and several other dermatological conditions [230]. Metabolites produced by gut bacteria (such as SCFAs like butyrate and neurotransmitters like 5-HT) travel through the bloodstream to directly regulate skin cell function, barrier integrity, and inflammation. Dysbiosis of the gut microbiota weakens the gut barrier, allowing harmful bacterial toxins and inflammatory markers to enter the bloodstream, directly promoting skin disorders. In addition to metabolites from the gut microbiota that pass through the gut–skin axis, cutaneous innate and adaptive immune responses can modulate the skin microbiota, but the gut microbiota also functions in educating the immune system [231].

The skin, which therefore harbors its own microbiome, is an ecosystem composed of 1.8 m^2^ of varied habitats with a profusion of creases, invaginations and specialized niches that sustain a broad variety of microorganisms, including bacteria, archaea, fungi, viruses and, unique to the integumentary system, *Demodex* mites [232]. These microscopic, eight-legged arachnids comprise a normal part of the flora inhabiting the skin and hair follicles. *Demodex folliculorum* and *Demodex brevis* are two species typically found on humans [233]. It is speculated that *Demodex* mites may play a role in the gut–skin axis by acting as both carriers of inflammatory bacteria and interacting with the host’s immune and digestive systems. Disruptions in the skin microbiome, involving *Propionibacterium acnes* and *Malassezia* yeast (principally *Malassezia restricta* and *Malassezia globosa* [234]), can alter the sebum composition of hair follicles and impact *Demodex* growth. *Demodex* carry bacteria on their surface, such as *Staphylococcus aureus*, *Staphylococcus epidermidis*, *Acinetobacter baumannii*, *Streptococcus pneumoniae*, *Klebsiella oxytoca*, and *Bacillus* spp. [235]. In addition, *Demodex* mites harbor *Bacillus oleronius* in their abdominal cavities [236]. Through neutrophil activation, these bacteria most likely act as an exacerbating element in the development of inflammatory processes [237]. The gut microbiome can influence *Demodex* infestation due to systemic inflammation as a result of dysbiosis. *Demodex folliculorum*, together with *Bacillus oleronius*, *Staphylococcus epidermidis*, and *Cutibacterium acnes*, has been implicated in the pathogenesis of rosacea, a chronic inflammatory skin disease [238]. An association between rosacea and numerous inflammatory gastrointestinal disorders has been reported. It is therefore possible that gut dysbiosis drives rosacea through the gut–skin axis, especially as treatments targeting dysbiosis of both the cutaneous and gastrointestinal microbiota have proven efficacious [239].

The current state of research on mammalian skin-associated archaea is limited, with the few papers focusing on potential skin archaeal communities often in disagreement with each other. The relative abundance of sequences linked to archaeal amplicon sequence variants were less than 1% for most mammalian skin samples and did not exceed 2% for any samples [240]. In another study, Thaumarchaeota archaea made up to 4.2% of the prokaryotic microbiome on healthy human skin, indicating they are common inhabitants. The study revealed that these skin-associated archaea possess the metabolic potential to oxidize ammonia, suggesting a specialized role in skin metabolism These archaea are also not methanogens, as in the colon, which is to be expected as skin is an aerobic environment [241].

Skin is second only to the gut in terms of bacterial density, with an approximate concentration of 10^4^ to 10^6^ bacteria per cm^2^, with over 200 genera reported using 16S rRNA amplicon sequencing [242]. The four dominant phyla of the skin microbiome are Actinomycetota (51.8%), Bacillota (24.4%), Pseudomonadota (16.5%) and Bacteroidota (6.3%). The principal role of the skin microbiome is threefold. First, there is resistance to pathogen colonization. Commensal microbes physically block harmful invaders. For example, *Staphylococcus epidermidis* produces antimicrobial peptides, such as the phenol-soluble modulins like PSM γ-/δ-toxins that actively suppress dangerous pathogens like *Staphylococcus aureus* [243]. As such, *Staphylococcus epidermidis* is capable of controlling inflammation, supporting antimicrobial defenses, and stabilizing the cutaneous barrier [244]. Second, there is immune education and modulation. The skin microbiome constantly communicates with the immune system to keep it functioning properly [245]. For example, the skin microbiota interacts with T cells to train them to recognize and respond aggressively to dangerous pathogens. In addition, beneficial bacteria release signaling molecules that reduce inflammaging and prevent autoimmune flare-ups [231,246]. Third, there is barrier maintenance and healing. The skin microbiome directly strengthens the physical structure of the skin [247]. Commensal bacteria secrete enzymes that convert skin lipids into ceramides, keeping the skin hydrated. Skin lipids (such as glucosylceramides and sphingomyelin) are converted into mature ceramides by hydrolytic enzymes. The primary enzymes responsible for this conversion are β-glucocerebrosidase and acid sphingomyelinase [248]. Although these two enzymes are effectively host-derived and produced by human keratinocytes in the epidermis [249], the skin microbiota, in particular *Staphylococcus epidermidis*, possesses functional analogs, specifically, bacterial sphingomyelinases and generic β-glucosidases that actively mirror or complement these host enzymatic activities [250]. This cooperative metabolic action actively reinforces the stratum corneum lipid matrix, directly preventing trans-epidermal water loss and aging [250]. The interaction of the skin microbiome, involving multiple bacterial species, and numerous lipid pathways has been demonstrated in a metagenomics and lipidomics investigation of a small cohort of 57 healthy individuals [251]. Finally, in addition to these three principal roles of the skin microbiome, commensal microbes like *Cutibacterium* spp. and *Staphylococcus* spp. release SCFAs. These keep skin pH as low as 4.5 in the outer stratum corneum [252], thereby preventing pathogen colonization, and regulating keratinocyte differentiation and lipid synthesis, which are essential for maintaining skin hydration and integrity [246].

In the gut–skin axis, the skin microbiome communicates with the gut through several key pathways. First is by immune cell migration. Inflammatory signals (such as cytokines) released by skin microbes travel through the lymphatic system and bloodstream, “educating” or activating systemic immune cells. These immune cells often migrate to the gut, impacting intestinal inflammation and immune tolerance [226,253]. Second is by the release of microbial byproducts and toxins. When skin barrier integrity is broken, microbial antigens (like LPS or endotoxins) can breach the systemic circulation. This triggers widespread, low-grade systemic inflammation that negatively impacts gut function and alters the intestinal microbiome [225]. Third is by vitamin D synthesis. The skin microbiome works in tandem with host cells to process UV-B (290–315 nm), synthesizing vitamin D. This hormone travels to the gut, where it helps maintain intestinal barrier integrity and supports a balanced gut microbiome. However, the active hormone is 1α,25-dihydroxy-vitamin D3, produced serially by the skin, liver, and kidney [254]. Fourth is by production of neuroendocrine molecules. The skin and its microbiota produce various neurotransmitters, stress hormones (like cortisol), and neuropeptides. When circulated to the gut, these signaling molecules can alter intestinal permeability and affect gut motility [255].

Clinical trial data on the gut–skin axis exist, but quality and depth vary by condition. Robust randomized controlled trials and systematic reviews mostly support oral probiotics for atopic dermatitis (eczema), while data for acne, rosacea, and psoriasis rely on smaller or more preliminary studies [256].

Therefore, the gut–skin axis is truly bidirectional and uses metabolic and immunological means for communication. It is now clear that the health of the skin and of the gut are closely linked. These are the two organs with the maximum exposure to the external environment and it is hardly surprising that they are the most highly populated with commensal organisms and have multiple means of communication via the gut–skin axis as described.

#### 3.1.7. Metabolite Signaling from the Gut Microbiota to the Oral Cavity

Through the gut–oral axis, the gut microbiota communicates by exchanging microbes and chemical signals through the bloodstream and immune system. For example, gut dysbiosis can trigger systemic inflammation, which weakens the body’s immune defenses and encourages the growth of periodontal pathogens in the mouth [257]. In the other direction, studies have shown a relationship between oral bacteria and gut dysbiosis. In one particular example, elderly persons with low and high periodontal inflammation had different enrichment of bacterial genera in the oral cavity. A low periodontal area of inflammation harbored *Lactobacillus*, *Roseburia,* and *Ruminococcus*. A high periodontal area of inflammation had *Coprococcus*, *Clostridiales*, and *Atopobium*. The degree of periodontal inflammation was associated with levels of gut dysbiosis and reduced levels of SCFA-producing bacteria [258]. In another example, dysbiosis of the oral microbiome, impelled by pathogens like *Porphyromonas gingivalis*, *Fusobacterium nucleatum*, *Streptococcus* spp., and *Helicobacter pylori*, disordered gut ecology by a number of processes, including direct translocation to the gut through the enteral route [259]. Certain metabolites, including SCFAs, TMAO, indole and its derivatives, secondary bile acids, and LPS produced by both oral and gut microbes may play a central role in facilitating gut–oral interactions [260].

The communication pathways from the gut to the oral cavity are threefold. First, through the systemic circulation. Gut microbial metabolites, such as SCFAs, LPS, and secondary bile acids are released into the circulation then travel through the bloodstream to directly influence oral immune responses and tissue health [260]. Second is immune cell modulation. Immune cells primed by gut bacteria circulate and alter the inflammatory environment inside the oral cavity, directly affecting gum health [261]. Third is fecal–oral transmission. Microorganisms can also physically transfer from the gut to the mouth via the fecal–oral route, especially within poor sanitary conditions, reshaping the oral microbial ecosystem [260]. In addition, when there is a change in the ratio between the Bacillota and Bacteroidota phyla, this promotes the activation of systemic inflammatory pathways, such as Th17 cell responses, which circulate and exacerbate oral inflammation [259]. The mouth and gut continuously exchange microbes. Under dysbiotic conditions, gut microbes can translocate to the oral cavity, altering the resident oral microbiome and contributing to local pathogenesis [262].

One unique aspect of the gut–oral axis is the presence of nitrate reducing bacteria that convert nitrate (NO_3_^−^) to nitrite (NO_2_^−^) in the oral cavity, leading to production of nitric oxide (NO^•^) [263,264]. Circulating dietary nitrate from the gut–oral axis is transported by sialin (SLC17A5) to concentrate nitrate in saliva at ten times its concentration in plasma, and up to 100 times more after a nitrate-rich meal [264], reaching concentrations up to 8 mM [264,265]. The oral bacteria that can execute the reduction in nitrate into nitrite include the genera *Neisseria*, *Rothia*, *Veillonella*, *Actinomyces*, *Corynebacterium*, *Haemophilus*, and *Kingella* [264,266,267]. When saliva is swallowed, the acidic gastric environment reduces nitrite to NO [268]. The nitrate–nitrite–NO pathway is seen as an alternative to the classical L-arginine–NO pathway as a means of NO production [269]. Overall, 25% of absorbed dietary nitrate is re-secreted in saliva, and 30% of this is reduced to nitrite by oral cavity bacteria. Since nitric oxide is a short half-life free radical and a gas, it does not pass down the gastrointestinal tract to the colonic microbiome. In the caecum and colon, *Lactobacillus* spp. and *Bifidobacterium* spp. are able to convert unabsorbed nitrate and nitrite to nitric oxide [270]. The nitrate–nitrite–nitric oxide reductive pathway of the oral cavity is exclusive to the gut–oral axis.

#### 3.1.8. Metabolite Signaling from the Gut Microbiota to Bone

The gut–bone axis is a biological pathway that links the gut microbiome, the immune system, and bone remodeling. Through immune signaling, inflammation regulation, and nutrient utilization, the gut helps influence how bone is broken down and rebuilt over time. Alterations in bone mineral density (BMD) can influence gut microbial composition. Conversely, microbial dysbiosis disrupts bone homeostasis through multiple pathways, including microbial metabolites, immune regulation, and neuroendocrine signaling. SCFAs suppress osteoclast differentiation and enhance intestinal calcium absorption, while gut dysbiosis promotes bone loss by impairing intestinal barrier integrity and increasing pro-inflammatory cytokines such as TNF-α and IL-6 [271]. Osteoporosis, characterized by reduced BMD and increased fracture risk, is closely linked to the gut microbiota, especially with relation to diet [272]. Moreover, a Mediterranean diet has been reported to improve bone health and reduced fracture risk [273]. Diet clearly influences gut microbiota composition, with Western diets enriching Pseudomonadota and depleting SCFA-producing bacteria. Conversely, plant-rich diets promote beneficial genera such as *Ruminococcus* and *Roseburia* that make SCFAs. For example, *Ruminococcus bromii* and *Ruminococcus hydrogenotrophicus* both synthesize high amounts of acetate from the complex polysaccharides present in a plant-rich diet [274]. *Roseburia intestinalis* and *Roseburia inulinivorans* synthesize both acetate and butyrate [275]. These SCFAs, enhanced in production by a plant-rich diet, pass through the gut–bone axis and benefit bone health by increasing bone mass and density. They do this primarily by inhibiting osteoclasts while simultaneously promoting the activity of osteoblasts [276,277]. But when the gut microbiota suffers dysbiosis due to prolonged consumption of a Western low-fiber diet, the Pseudomonadota genera such as *Escherichia* and *Salmonella* are increased, and these produce LPS, which translocates into the circulation through a compromised intestinal barrier [272]. The resulting systemic inflammation drives osteoclastogenesis and bone loss [278,279]. Dysbiotic Pseudomonadota bacteria such as *Shigella* spp. convert dietary choline and phospholipids into TMA, subsequently metabolized in the liver to TMAO [280]. Elevated plasma TMAO is related negatively to BMD and positively to TNF-α concentration, directly associating TMAO with inflammation-driven osteoporosis [281].

A significant negative correlation exists between plasma TMAO and bone mineral density (BMD), coupled with a robust positive correlation with serum TNF-α concentrations. This directly positions TMAO as a critical mediator linking gut microbiota dysbiosis to inflammation-driven osteoporosis. Accumulating evidence suggests that TMAO compromises bone homeostasis through divergent cellular pathways affecting both bone resorption and formation. On one hand, TMAO exacerbates bone resorption by enhancing the secretion of pro-inflammatory cytokines, such as TNF-α and IL-6, which subsequently activate the canonical NF-κB signaling cascade to promote osteoclast differentiation and functional maturation. On the other hand, the recent literature underscores the detrimental impact of TMAO on bone formation. Elevated systemic TMAO triggers profound endoplasmic reticulum stress within bone-forming cells, selectively upregulating the PERK-ATF4 signaling axis to induce osteoblast cell-cycle arrest and apoptosis. Consequently, TMAO shifts the bone remodeling equilibrium toward net bone loss by simultaneously driving NF-κB-mediated osteoclastogenesis and accelerating PERK-ATF4-mediated osteoblast depletion [272,282].

Bones are a particular common site for metastasis due to a plentiful supply of growth and neovascularization factors and a plentiful blood flow. This mainly affects patients with thyroid, prostate, breast, lung, and kidney cancers. The gut dysbiosis-derived disruption in the delicate balance of bone modeling and remodeling may initiate a favorable environment for metastatic growth. The role of the gut microbiome in bone metastasis development has been discussed in detail [283].

While a lot of research focuses on how the microbiome affects the skeleton, bones also signal back to the gut. Skeletal health and the microbiome influence each other through several pathways. First, bones actively secrete hormones like osteocalcin. In return, these bone-derived hormones help regulate energy metabolism and insulin sensitivity, which directly impacts the gut environment [284]. Second, the skeleton contains bone marrow, the primary site for immune cell production. Bones release factors that influence immune cell development. These immune cells then migrate to the gut, where they dictate how the body responds to gut bacteria [285]. Third, the gut absorbs calcium and phosphorus, essential for bone homeostasis. When bone resorption occurs, it releases stored minerals and signaling molecules that can alter systemic homeostasis [285].

#### 3.1.9. Metabolite Signaling from the Gut Microbiota to the Heart

The gut microbiota constantly signals to the heart through a complex bidirectional pathway called the gut–heart axis [286]. The gut microbiome produces chemical messengers that enter your bloodstream, directly regulating cardiovascular function, inflammation, and heart health. As stated above, trimethylamine (TMA), which is further metabolized in the liver to TMAO, is strongly associated with plaque buildup in arteries and an increased risk of coronary heart disease. Conversely, the microbiota also ferment dietary fiber to produce beneficial SCFAs, which help lower blood pressure and reduce inflammation. Therefore, the key microbial metabolites, such as SCFAs, TMAO, and LPS have been implicated in mechanisms involving endothelial, cardiac fibroblast, cardiomyocyte dysfunction, systemic inflammation, and metabolic dysregulation [287]. Dysbiosis is linked to various intestinal diseases, including inflammatory bowel disease (IBD) and irritable bowel syndrome (IBS) [288]. Recent research provided evidence that IBD is associated with cardiovascular diseases (CVDs) such as coronary artery disease, atrial fibrillation, stroke, and heart failure. Mechanisms of these associations included atherosclerosis/endothelial dysfunction, dyslipidemia, thrombocytosis, and dysbiosis of the gut microbiota [289]. During dysbiosis, epithelial barrier permeability increases, permitting the exit of bacterial toxins such as LPS into the circulation where they act on Toll-like receptors, leading to heightened inflammatory signaling [286].

The gut microbiota-dependent metabolite phenylacetylgutamine (PAGln) is both associated with atherothrombotic heart disease in humans and its murine equivalent, phenylacetylglycine (PAGly), is mechanistically linked to cardiovascular disease pathogenesis in mouse models via modulation of adrenergic receptor signaling [290]. PAGln is clinically and mechanistically linked to the presence and severity of heart failure (HF). Modulating the gut microbiome, in general, and PAGln production, in particular, may represent a potential therapeutic target for modulating HF [290,291].

Cardiac arrhythmias are very common causes of morbidity and mortality in humans, the mechanisms of which are neither completely understood nor explained by traditional risk factors. Dysregulation by the gut microbiome of the immune and inflammatory pathways affecting the autonomic nervous system is presumed to contribute to cardiac arrhythmias, and this has been established for atrial fibrillation (AF). Activation of the cardiac autonomic nervous system appeared to be the root cause of AF, with gut microbiome-derived TMAO facilitating its progression [292].

Accumulated data reveals that the gut microbiota and its metabolites regulate myocardial health through intricate cross-organ networks, including the gut–brain–heart, gut–liver–heart, and gut–lung–heart axes. These findings suggest that the heart plays a vital role in systemic host–microbe communication [293].

#### 3.1.10. Signal Strength and Tissue Sensitivity

What determines how much of a gut microbiota signaling metabolite reaches a particular distal organ is a complex matter. These issues can be illustrated using the SCFA acetate as an example because microbiota generated acetate should in theory be able to reach most organs perfused by the general blood circulation. Acetate produced by fermentation of dietary fiber is absorbed across the colonic mucosa via passive diffusion of the non-ionic form or active transport of the ionic form via monocarboxylate transporters [294], such as MCT1 (SLC16A1) and SLC5A8. From the portal vein, a proportion of acetate is extracted by the liver depending on the portal venous acetate concentration, when the acetate concentration exceeds 180 µM. Moreover, acetate release by the liver into the hepatic vein was highly statistically and linearly correlated with the portal vein acetate concentration. It appeared in these early investigations in the rat and in patients with cirrhosis that the liver regulated the systemic acetate concentration in both man and rat [295]. Systemic arterial concentrations of acetate typically range between 60 µM and 200 µM, representing a diluted pool available to peripheral tissues like skeletal muscle, adipose tissue, brain, heart, lung, kidney and bone marrow.

The question remains what determines how much acetate is taken up by each peripheral organ. It can be readily calculated that acetate in arterial blood at pH ~7.40 will be only 0.23% unionized and 99.77% ionized. Cell membranes are impermeable to metabolic anions such as acetate, which require MCT transporters to enter tissues [296]. MCT1 is ubiquitously expressed but particularly in oxidative, high-energy-demand organs, such as brain, heart, and skeletal muscle [297,298]. MCT1 is expressed in the capillary endothelial cells of the BBB [299,300] and is expected to facilitate access of acetate to the brain.

**Figure 6:**
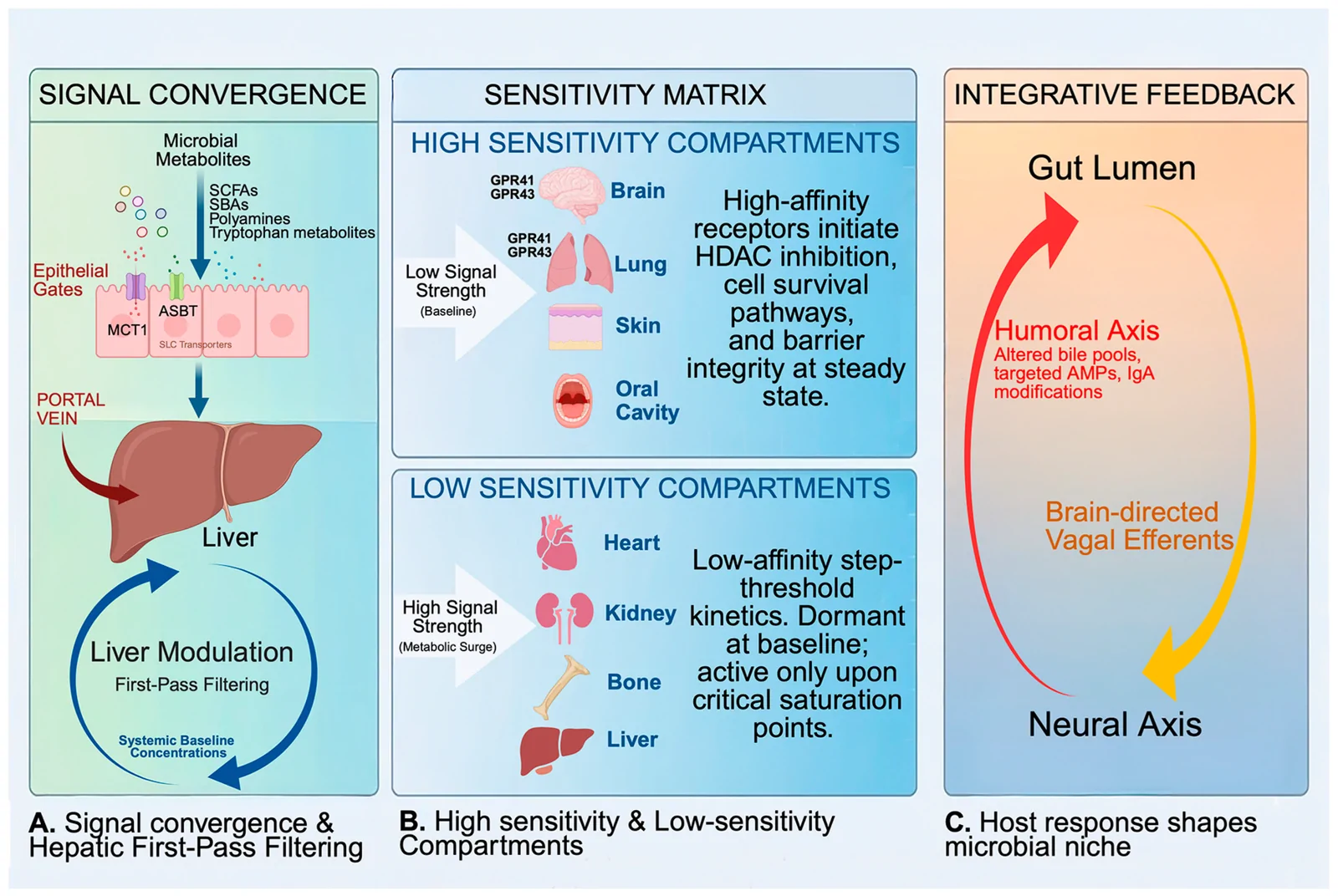
(**A**) The microbial production of metabolite signaling molecules, their passage through gut epithelial cells into the hepatic portal vein to the liver for first-pass filtering, generating baseline concentrations for systemic distribution. (**B**) Target organ uptake determined by high-affinity receptors in brain, lung, skin and oral cavity, even when the metabolite signal is low. Target organ uptake by heart, kidney, bone, and liver determined by a high metabolite signal but critical saturation triggers uptake. (**C**) Tissue response, for example by liver (altered bile acid pools) and immune cells (antimicrobial peptides (AMPs) and modified IgAs)), shapes the gut microbiome through the humoral axis, while the brain shapes the gut microbiome via the vagus nerve.

These principles of signal strength and tissue sensitivity for acetate are expected to apply in general terms for the other gut microbiome signaling metabolites, including other SCFAs, tryptophan metabolites, the polyamines, and secondary bile acids described above. A comprehensive mechanism overview is given in Figure 6.

#### 3.1.11. Cooperative Strategies Between Signaling Metabolites

Microbiome metabolites cooperate at target organs via systemic co-metabolism, receptor agonism, and cross-feeding. They travel through blood or portal circulation to co-regulate host immunity, metabolic homeostasis, and neurological signaling at distant sites. Rather than acting alone, distinct chemical families like SCFAs, tryptophan catabolites, and secondary bile acids work together. They use cooperative biochemical strategies to control a single organ’s function. For example, SCFAs bind to GPR41/43 receptors on the surface of BBB cells, signaling downstream to support the physical structure of the barrier [301]. Then, indoles cross the barrier and directly enter the cytoplasm of microglia cells to bind to AhR [302]. Together, the surface signal (SCFAs) and the internal signal (indoles) lock brain immune cells into an anti-inflammatory state. Another example in the kidney concerns the tryptophan metabolite and protein-bound uremic toxin IS and SCFAs that compete for the same host transport and clearance systems. IS can cause kidney fibrosis if it accumulates. SCFAs propionate and butyrate upregulate the transporter SLC22A6 (organic anion transporter 1) on the basolateral side of renal proximal tubule cells by the inhibition of HDACs. Using a kidney-on-chip system, it was demonstrated that butyrate exposure directly accelerated the secretion and clearance of IS into the luminal compartment for urinary excretion [303]. In this way, SCFAs balance out the toxic stress caused by tryptophan byproducts at the kidney tissue level, maintaining a stable renal filtration rate.

## 4. Conclusions

“All disease begins in the gut.”

(Hippocrates)

“It is literally the engine for everything… The neurosurgeons think it’s the brain, but it’s the gut.”

(Dr. Robynne Chutkan)

Deconstructing Hippocrates’ quotation requires consideration of the origin of the word diet, from the Greek word ίαιτα (diaita). In Hippocrates’ time (460–370 BCE), diet had a much broader meaning than how we use it today. It represented a notion of a healthy lifestyle including both mental and physical health, rather than the narrow meaning of a weight-loss regimen [304]. Hippocrates therefore meant that both mental and physical health began in the gut. Obviously, he had no knowledge of bacteria or the gut microbiome.

We now know that the gut microbiota comprises approximately 38 trillion microorganisms [305]. “People talk about trillion all the time these days, especially with regard to budgets and so on. But if you count one number per second, it will take you 11 days to count to a million, 31 years to count to a billion, and 31,000 years to count to a trillion. If only people knew how big [a] trillion is, they’d probably appreciate how many microbiota we have in our body” [306]. As Dr. Chutkan states, it [the gut] is literally the engine for everything and, as we have described in this review, the gut microbiome, as a hub, signals to the gut epithelium, the brain, the lungs, the liver, the kidneys, the skin, the oral cavity, the skeleton, and the heart. In addition, the gut microbiome signals to adipose tissue through the gut–adipose axis using SCFAs and secondary bile acids [307] and, in addition, to skeletal muscle though the gut–muscle axis [308]. Figure 2 (SCFAs), Figure 3 (TRPMs), Figure 4 (polyamines), and Figure 5 (secondary bile acids) catalog the individual metabolic messengers released by the gut microbiome. A total of 18 small signaling molecules are described and there are unquestionably many more, and undoubtedly many more target tissues affected by the gut microbiome than we have discussed here.

Both the normal physiology and pathophysiology of the host is under the direction of the gut microbiome, acting like a conductor of a large orchestra. The signaling molecules emanating from the gut microbiome may be potential indicators and biomarkers for both wellness and sickness. As we have similarly stated elsewhere [309], it might be predicted that clinical laboratories in the future will assay patient samples for both beneficial and deleterious microbiota metabolites as biomarkers of both health and disease risk.

## Data Availability

No new data were created or analyzed in this study. Data sharing is not applicable to this article.

## Abbreviations

The following abbreviations are used in this manuscript:

SCFA	Short-Chain Fatty Acid
TRPM	Tryptophan Metabolite
TLR	Toll-Like Receptor
Tregs	Regulatory T Cells
CGRP	Calcitonin Gene-Related Peptide
BCEC	Brain Capillary Endothelial Cell
TMA	Trimethylamine
TMAO	Trimethylamine N-Oxide
HDAC	Histone Deacetylase
BBB	Blood–Brain Barrier
IE	Indole-3-Ethanol (Tryptophol)
IPA	Indole-3-Propionic Acid
ILA	Indole-3-Lactic Acid
IAA	Indole-3-Acetic Acid
IAld	Indole-3-Aldehyde
IA	Indole-3-Acrylic Acid
AMD1	S-Adenosylmethionine Decarboxylase 1
DCA	Deoxycholic Acid
LCA	Lithocholic Acid
UDCA	Ursodeoxycholic Acid
HDCA	Hyodeoxycholic Acid
AMPK	AMP-Activated Protein KinaseAhR, Aryl Hydrocarbon Receptor
FXR	Farnesoid X Receptor
PCS	P-Cresyl Sulfate
CKD	Chronic Kidney Disease
LPS	Lipopolysaccharides
BMD	Bone Mineral Density
IBD	Inflammatory Bowel Disease
IBS	Irritable Bowel Syndrome
CVD	Cardiovascular Disease
HF	Heart Failure
AF	Atrial Fibrillation

## References

[1] Saxami G., Kerezoudi E.N., Eliopoulos C., Arapoglou D., Kyriacou A. (2023). The Gut-Organ Axis within the Human Body: Gut Dysbiosis and the Role of Prebiotics. Life.

[2] Ahlawat S., Asha N., Sharma K.K. (2021). Gut-organ axis: A microbial outreach and networking. Lett. Appl. Microbiol..

[3] Motataianu A., Serban G., Andone S. (2023). The Role of Short-Chain Fatty Acids in Microbiota-Gut-Brain Cross-Talk with a Focus on Amyotrophic Lateral Sclerosis: A Systematic Review. Int. J. Mol. Sci..

[4] Appleton J. (2018). The Gut-Brain Axis: Influence of Microbiota on Mood and Mental Health. Integr. Med..

[5] Shen H., Wang S.Y., Zhao Y.Y., Zhou J.L., Zhao J., Zhu W.K. (2026). Brain-gut-microbiota axis: A review on the bidirectional regulatory mechanisms between gut microbiota and brain and their disease interactions. Front. Microbiol..

[6] Beyoglu D., Park E.J., Quinones-Lombrana A., Dave A., Parande F., Pezzuto J.M., Idle J.R. (2022). Addition of grapes to both a standard and a high-fat Western pattern diet modifies hepatic and urinary metabolite profiles in the mouse. Food Funct..

[7] Pezzuto J.M., Dave A., Park E.J., Beyoglu D., Idle J.R. (2022). Short-Term Grape Consumption Diminishes UV-Induced Skin Erythema. Antioxidants.

[8] Beyoglu D., Idle J.R. (2025). The Microbiome and Metabolic Dysfunction-Associated Steatotic Liver Disease. Int. J. Mol. Sci..

[9] Beyoglu D., Idle J.R. (2022). The gut microbiota–A vehicle for the prevention and treatment of hepatocellular carcinoma. Biochem. Pharmacol..

[10] Beyoglu D., Idle J.R. (2026). The Gut-Lung Microbiome Crosstalk and Pulmonary Disease. Biomolecules.

[11] Almeida C., Goncalves-Nobre J.G., Alpuim Costa D., Barata P. (2023). The potential links between human gut microbiota and cardiovascular health and disease–is there a gut-cardiovascular axis?. Front. Gastroenterol..

[12] Lin L., Xiang S., Chen Y., Liu Y., Shen D., Yu X., Wu Z., Sun Y., Chen K., Luo J. (2024). Gut microbiota: Implications in pathogenesis and therapy to cardiovascular disease (Review). Exp. Ther. Med..

[13] Tsuji K., Uchida N., Nakanoh H., Fukushima K., Haraguchi S., Kitamura S., Wada J. (2024). The Gut-Kidney Axis in Chronic Kidney Diseases. Diagnostics.

[14] Jin Y., Zhang S.J., Zhuang S., Li P., Miao H., Zhao Y.Y. (2026). Microbiota-gut-kidney axis in health and renal disease. Int. J. Biol. Sci..

[15] Mahmud M.R., Akter S., Tamanna S.K., Mazumder L., Esti I.Z., Banerjee S., Akter S., Hasan M.R., Acharjee M., Hossain M.S. (2022). Impact of gut microbiome on skin health: Gut-skin axis observed through the lenses of therapeutics and skin diseases. Gut Microbes.

[16] De Pessemier B., Grine L., Debaere M., Maes A., Paetzold B., Callewaert C. (2021). Gut-Skin Axis: Current Knowledge of the Interrelationship between Microbial Dysbiosis and Skin Conditions. Microorganisms.

[17] Gao T., Wang X., Li Y., Ren F. (2023). The Role of Probiotics in Skin Health and Related Gut-Skin Axis: A Review. Nutrients.

[18] Indrio F., Salatto A. (2025). Gut Microbiota-Bone Axis. Ann. Nutr. Metab..

[19] Zaiss M.M., Jones R.M., Schett G., Pacifici R. (2019). The gut-bone axis: How bacterial metabolites bridge the distance. J. Clin. Investig..

[20] Li R., Miao Z., Liu Y., Chen X., Wang H., Su J., Chen J. (2024). The Brain-Gut-Bone Axis in Neurodegenerative Diseases: Insights, Challenges, and Future Prospects. Adv. Sci..

[21] Sumida K., Kovesdy C.P. (2019). The gut-kidney-heart axis in chronic kidney disease. Physiol. Int..

[22] Yang T., Richards E.M., Pepine C.J., Raizada M.K. (2018). The gut microbiota and the brain-gut-kidney axis in hypertension and chronic kidney disease. Nat. Rev. Nephrol..

[23] Zildzic M., Salihefendic N., Masic I. (2024). The Mesentery’s Pivotal Role in the Brain-Gut-Liver Axis. Med. Arch..

[24] Guo Z., Yang J., Zang R., Yang Y., Wang Q., Xu C. (2026). The brain-gut-skin axis in inflammatory and disfiguring skin diseases: Mechanistic insights, clinical correlations, and therapeutic strategies. Front. Immunol..

[25] Vardanyan G.S., Harutyunyan H.S., Aghajanov M.I., Vardanyan R.S. (2020). Neurochemical regulators of food behavior for pharmacological treatment of obesity: Current status and future prospects. Future Med. Chem..

[26] Ansari A., Bose S., Yadav M.K., Wang J.H., Song Y.K., Ko S.G., Kim H. (2016). CST, an Herbal Formula, Exerts Anti-Obesity Effects through Brain-Gut-Adipose Tissue Axis Modulation in High-Fat Diet Fed Mice. Molecules.

[27] Liu T., Wu H., Wei J. (2026). Beyond the Brain: Exploring the multi-organ axes in Parkinson’s disease pathogenesis. J. Adv. Res..

[28] Feng C., He Q., Kwok L.Y., Sun Z. (2026). Gut microbiota-derived metabolites as systemic messengers: Mechanisms of gut-organ communication and translational opportunities. Trends Food Sci. Technol..

[29] Lin X., Yu Z., Liu Y., Li C., Hu H., Hu J.C., Liu M., Yang Q., Gu P., Li J. (2025). Gut-X axis. Imeta.

[30] Farre-Maduell E., Casals-Pascual C. (2019). The origins of gut microbiome research in Europe: From Escherich to Nissle. Hum. Microbiome J..

[31] Sekirov I., Russell S.L., Antunes L.C., Finlay B.B. (2010). Gut microbiota in health and disease. Physiol. Rev..

[32] Moszak M., Szulinska M., Bogdanski P. (2020). You Are What You Eat-The Relationship between Diet, Microbiota, and Metabolic Disorders-A Review. Nutrients.

[33] Frank D.N., St Amand A.L., Feldman R.A., Boedeker E.C., Harpaz N., Pace N.R. (2007). Molecular-phylogenetic characterization of microbial community imbalances in human inflammatory bowel diseases. Proc. Natl. Acad. Sci. USA.

[34] Xu J., Gordon J.I. (2003). Honor thy symbionts. Proc. Natl. Acad. Sci. USA.

[35] Leonard J.M., Toro D.D. (2023). Defining the Microbiome Components (Bacteria, Viruses, Fungi) and Microbiome Geodiversity. Surg. Infect..

[36] Shen J., Zhang J., Mo L., Li Y., Li Y., Li C., Kuang X., Tao Z., Qu Z., Wu L. (2023). Large-scale phage cultivation for commensal human gut bacteria. Cell Host Microbe.

[37] Duller S., Moissl-Eichinger C. (2024). Archaea in the Human Microbiome and Potential Effects on Human Infectious Disease. Emerg. Infect. Dis..

[38] Harris S.C., Devendran S., Mendez-Garcia C., Mythen S.M., Wright C.L., Fields C.J., Hernandez A.G., Cann I., Hylemon P.B., Ridlon J.M. (2018). Bile acid oxidation by Eggerthella lenta strains C592 and DSM 2243(T). Gut Microbes.

[39] Kuehnast T., Kumpitsch C., Mohammadzadeh R., Weichhart T., Moissl-Eichinger C., Heine H. (2025). Exploring the human archaeome: Its relevance for health and disease, and its complex interplay with the human immune system. FEBS J..

[40] Zhang L., Zhan H., Xu W., Yan S., Ng S.C. (2021). The role of gut mycobiome in health and diseases. Ther. Adv. Gastroenterol..

[41] Carrillo-Serradell L., Liu-Tindall J., Planells-Romeo V., Aragon-Serrano L., Isamat M., Gabaldon T., Lozano F., Velasco-de Andres M. (2025). The Human Mycobiome: Composition, Immune Interactions, and Impact on Disease. Int. J. Mol. Sci..

[42] Gaspar B.S., Rosu O.A., Enache R.M., Manciulea Profir M., Pavelescu L.A., Cretoiu S.M. (2025). Gut Mycobiome: Latest Findings and Current Knowledge Regarding Its Significance in Human Health and Disease. J. Fungi.

[43] Belvoncikova P., Splichalova P., Videnska P., Gardlik R. (2022). The Human Mycobiome: Colonization, Composition and the Role in Health and Disease. J. Fungi.

[44] Huang H., Wang Q., Yang Y., Zhong W., He F., Li J. (2024). The mycobiome as integral part of the gut microbiome: Crucial role of symbiotic fungi in health and disease. Gut Microbes.

[45] Chabe M., Lokmer A., Segurel L. (2017). Gut Protozoa: Friends or Foes of the Human Gut Microbiota?. Trends Parasitol..

[46] Dubik M., Pilecki B., Moeller J.B. (2022). Commensal Intestinal Protozoa-Underestimated Members of the Gut Microbial Community. Biology.

[47] Burgess S.L., Gilchrist C.A., Lynn T.C., Petri W.A. (2017). Parasitic Protozoa and Interactions with the Host Intestinal Microbiota. Infect. Immun..

[48] Kim M.J., Lee Y.J., Kim T.J., Won E.J. (2021). Gut Microbiome Profiles in Colonizations with the Enteric Protozoa Blastocystis in Korean Populations. Microorganisms.

[49] Wawrzyniak I., Poirier P., Viscogliosi E., Dionigia M., Texier C., Delbac F., Alaoui H.E. (2013). Blastocystis, an unrecognized parasite: An overview of pathogenesis and diagnosis. Ther. Adv. Infect. Dis..

[50] Gerrick E.R., Zlitni S., West P.T., Carter M.M., Mechler C.M., Olm M.R., Caffrey E.B., Li J.A., Higginbottom S.K., Severyn C.J. (2024). Metabolic diversity in commensal protists regulates intestinal immunity and trans-kingdom competition. Cell.

[51] Gerrick E.R., Howitt M.R. (2025). The Lost Kingdom: Commensal protists in the gut microbiota. Trends Microbiol..

[52] Ullah H., Arbab S., Tian Y., Chen Y., Liu C.Q., Li Q., Li K. (2024). Crosstalk between gut microbiota and host immune system and its response to traumatic injury. Front. Immunol..

[53] Salvo-Romero E., Stokes P., Gareau M.G. (2020). Microbiota-immune interactions: From gut to brain. LymphoSign J..

[54] Kiran S., Cruz A.R., Daniau A., Ma B., Marbouty M., Pipoli Da Fonseca J., Legrand A., Baudry L., Cokelaer T., Bensussan M. (2026). Segmented filamentous bacteria are worldwide human gut commensals. Nat. Commun..

[55] Yurtseven B., Aydemir E., Ayaz F. (2025). The Role of Intestinal Microbiota and Immune System Interactions in Autoimmune Diseases. Immunotargets Ther..

[56] Campbell C., Kandalgaonkar M.R., Golonka R.M., Yeoh B.S., Vijay-Kumar M., Saha P. (2023). Crosstalk between Gut Microbiota and Host Immunity: Impact on Inflammation and Immunotherapy. Biomedicines.

[57] Hidalgo-Cantabrana C., Delgado S., Ruiz L., Ruas-Madiedo P., Sanchez B., Margolles A. (2017). Bifidobacteria and Their Health-Promoting Effects. Microbiol. Spectr..

[58] Ruiz L., Delgado S., Ruas-Madiedo P., Sanchez B., Margolles A. (2017). Bifidobacteria and Their Molecular Communication with the Immune System. Front. Microbiol..

[59] Luo W., Li R., Pan C., Luo C. (2025). Gut microbiota-derived metabolites in immunomodulation and gastrointestinal cancer immunotherapy. Front. Immunol..

[60] Zegarra-Ruiz D.F., Kim D.V., Norwood K., Kim M., Wu W.H., Saldana-Morales F.B., Hill A.A., Majumdar S., Orozco S., Bell R. (2021). Thymic development of gut-microbiota-specific T cells. Nature.

[61] Clinton N.A., Hameed S.A., Agyei E.K., Jacob J.C., Oyebanji V.O., Jabea C.E. (2022). Crosstalk between the Intestinal Virome and Other Components of the Microbiota, and Its Effect on Intestinal Mucosal Response and Diseases. J. Immunol. Res..

[62] Yu X., Cheng L., Yi X., Li B., Li X., Liu X., Liu Z., Kong X. (2024). Gut phageome: Challenges in research and impact on human microbiota. Front. Microbiol..

[63] Emencheta S.C., Olovo C.V., Eze O.C., Kalu C.F., Berebon D.P., Onuigbo E.B., Vila M., Balcao V.M., Attama A.A. (2023). The Role of Bacteriophages in the Gut Microbiota: Implications for Human Health. Pharmaceutics.

[64] Cao Z., Sugimura N., Burgermeister E., Ebert M.P., Zuo T., Lan P. (2022). The gut virome: A new microbiome component in health and disease. EBioMedicine.

[65] Alkhalil S.S. (2023). The role of bacteriophages in shaping bacterial composition and diversity in the human gut. Front. Microbiol..

[66] Lusiak-Szelachowska M., Weber-Dabrowska B., Jonczyk-Matysiak E., Wojciechowska R., Gorski A. (2017). Bacteriophages in the gastrointestinal tract and their implications. Gut Pathog..

[67] Gorski A., Jonczyk-Matysiak E., Lusiak-Szelachowska M., Miedzybrodzki R., Weber-Dabrowska B., Borysowski J. (2018). Bacteriophages targeting intestinal epithelial cells: A potential novel form of immunotherapy. Cell Mol. Life Sci..

[68] Carroll-Portillo A., Lin H.C. (2019). Bacteriophage and the Innate Immune System: Access and Signaling. Microorganisms.

[69] Houshyar Y., Zhang F., Tavakoli P., Grimm M.C., Hold G.L. (2026). Neglected kingdoms: The gut virome, mycobiome and their role in inflammatory bowel disease. Gut Microbes.

[70] Alonso-Allende J., Milagro F.I., Aranaz P. (2024). Health Effects and Mechanisms of Inulin Action in Human Metabolism. Nutrients.

[71] Sabater-Molina M., Larque E., Torrella F., Zamora S. (2009). Dietary fructooligosaccharides and potential benefits on health. J. Physiol. Biochem..

[72] Mei Z., Yuan J., Li D. (2022). Biological activity of galacto-oligosaccharides: A review. Front. Microbiol..

[73] Lara-Espinoza C., Carvajal-Millan E., Balandran-Quintana R., Lopez-Franco Y., Rascon-Chu A. (2018). Pectin and Pectin-Based Composite Materials: Beyond Food Texture. Molecules.

[74] Okonkwo C.E., O’Connor M.J., Wong Y.L., Nystrom L., Al-Marzouqi A.H., Ayyash M., Boldor D., Kamal-Eldin A. (2026). Chemical and structural characterization of hemicellulose from date fruits (*Phoenix dactylifera* L.). Front. Nutr..

[75] Dave A., Park E.J., Kumar A., Parande F., Beyoglu D., Idle J.R., Pezzuto J.M. (2022). Consumption of Grapes Modulates Gene Expression, Reduces Non-Alcoholic Fatty Liver Disease, and Extends Longevity in Female C57BL/6J Mice Provided with a High-Fat Western-Pattern Diet. Foods.

[76] Dave A., Beyoglu D., Park E.J., Idle J.R., Pezzuto J.M. (2023). Influence of grape consumption on the human microbiome. Sci. Rep..

[77] Wang Z., Peters B.A., Yu B., Grove M.L., Wang T., Xue X., Thyagarajan B., Daviglus M.L., Boerwinkle E., Hu G. (2024). Gut Microbiota and Blood Metabolites Related to Fiber Intake and Type 2 Diabetes. Circ. Res..

[78] Morrison D.J., Preston T. (2016). Formation of short chain fatty acids by the gut microbiota and their impact on human metabolism. Gut Microbes.

[79] Rios-Covian D., Ruas-Madiedo P., Margolles A., Gueimonde M., de Los Reyes-Gavilan C.G., Salazar N. (2016). Intestinal Short Chain Fatty Acids and their Link with Diet and Human Health. Front. Microbiol..

[80] Yu X., Gurry T., Nguyen L.T.T., Richardson H.S., Alm E.J. (2020). Prebiotics and Community Composition Influence Gas Production of the Human Gut Microbiota. mBio.

[81] Dridi B., Henry M., El Khechine A., Raoult D., Drancourt M. (2009). High prevalence of Methanobrevibacter smithii and Methanosphaera stadtmanae detected in the human gut using an improved DNA detection protocol. PLoS ONE.

[82] Lange O., Proczko-Stepaniak M., Mika A. (2023). Short-Chain Fatty Acids-A Product of the Microbiome and Its Participation in Two-Way Communication on the Microbiome-Host Mammal Line. Curr. Obes. Rep..

[83] Park I., Mannaa M. (2025). Fermented Foods as Functional Systems: Microbial Communities and Metabolites Influencing Gut Health and Systemic Outcomes. Foods.

[84] Rowland I., Gibson G., Heinken A., Scott K., Swann J., Thiele I., Tuohy K. (2018). Gut microbiota functions: Metabolism of nutrients and other food components. Eur. J. Nutr..

[85] Jacquier E.F., van de Wouw M., Nekrasov E., Contractor N., Kassis A., Marcu D. (2024). Local and Systemic Effects of Bioactive Food Ingredients: Is There a Role for Functional Foods to Prime the Gut for Resilience?. Foods.

[86] Liu M., Lu Y., Xue G., Han L., Jia H., Wang Z., Zhang J., Liu P., Yang C., Zhou Y. (2024). Role of short-chain fatty acids in host physiology. Anim. Model Exp. Med..

[87] Deshpande O.A., Mohiuddin S.S. (2026). Biochemistry, Oxidative Phosphorylation. StatPearls.

[88] Kim C.H., Park J., Kim M. (2014). Gut microbiota-derived short-chain Fatty acids, T cells, and inflammation. Immune Netw..

[89] Thulasinathan B., Suvilesh K.N., Maram S., Grossmann E., Ghouri Y., Teixeiro E.P., Chan J., Kaif J.T., Rachagani S. (2025). The impact of gut microbial short-chain fatty acids on colorectal cancer development and prevention. Gut Microbes.

[90] Shin Y., Han S., Kwon J., Ju S., Choi T.G., Kang I., Kim S.S. (2023). Roles of Short-Chain Fatty Acids in Inflammatory Bowel Disease. Nutrients.

[91] Parada Venegas D., De la Fuente M.K., Landskron G., Gonzalez M.J., Quera R., Dijkstra G., Harmsen H.J.M., Faber K.N., Hermoso M.A. (2019). Short Chain Fatty Acids (SCFAs)-Mediated Gut Epithelial and Immune Regulation and Its Relevance for Inflammatory Bowel Diseases. Front. Immunol..

[92] Xiao J., Guo X., Wang Z. (2024). Crosstalk between hypoxia-inducible factor-1alpha and short-chain fatty acids in inflammatory bowel disease: Key clues toward unraveling the mystery. Front. Immunol..

[93] O’Riordan K.J., Collins M.K., Moloney G.M., Knox E.G., Aburto M.R., Fulling C., Morley S.J., Clarke G., Schellekens H., Cryan J.F. (2022). Short chain fatty acids: Microbial metabolites for gut-brain axis signalling. Mol. Cell Endocrinol..

[94] Liu J., Wang F., Liu S., Du J., Hu X., Xiong J., Fang R., Chen W., Sun J. (2017). Sodium butyrate exerts protective effect against Parkinson’s disease in mice via stimulation of glucagon like peptide-1. J. Neurol. Sci..

[95] Onyszkiewicz M., Gawrys-Kopczynska M., Konopelski P., Aleksandrowicz M., Sawicka A., Kozniewska E., Samborowska E., Ufnal M. (2019). Butyric acid, a gut bacteria metabolite, lowers arterial blood pressure via colon-vagus nerve signaling and GPR41/43 receptors. Pflug. Arch..

[96] Brand A., Richter-Landsberg C., Leibfritz D. (1997). Metabolism of acetate in rat brain neurons, astrocytes and cocultures: Metabolic interactions between neurons and glia cells, monitored by NMR spectroscopy. Cell Mol. Biol..

[97] Waniewski R.A., Martin D.L. (1998). Preferential utilization of acetate by astrocytes is attributable to transport. J. Neurosci..

[98] Dienel G.A., Cruz N.F. (2006). Astrocyte activation in working brain: Energy supplied by minor substrates. Neurochem. Int..

[99] Rowlands B.D., Klugmann M., Rae C.D. (2017). Acetate metabolism does not reflect astrocytic activity, contributes directly to GABA synthesis, and is increased by silent information regulator 1 activation. J. Neurochem..

[100] Peng F., Zhuang H., Yang M., Zhang Y., Tang X., Xin J., Yu H., Li T. (2026). Acetate enhances neuronal plasticity and reduces ischemic brain injury by promoting tau protein lactylation. J. Cereb. Blood Flow Metab..

[101] He Q., Ji L., Wang Y., Zhang Y., Wang H., Wang J., Zhu Q., Xie M., Ou W., Liu J. (2024). Acetate enables metabolic fitness and cognitive performance during sleep disruption. Cell Metab..

[102] Hoyles L., Snelling T., Umlai U.K., Nicholson J.K., Carding S.R., Glen R.C., McArthur S. (2018). Microbiome-host systems interactions: Protective effects of propionate upon the blood-brain barrier. Microbiome.

[103] Wu Q., Dong J., Bai X., Jiang Y., Li J., Fan S., Cheng Y., Jiang G. (2022). Propionate ameliorates diabetes-induced neurological dysfunction through regulating the PI3K/Akt/eNOS signaling pathway. Eur. J. Pharmacol..

[104] Gold A., Kaye S., Gao J., Zhu J. (2024). Propionate Decreases Microglial Activation but Impairs Phagocytic Capacity in Response to Aggregated Fibrillar Amyloid Beta Protein. ACS Chem. Neurosci..

[105] Chen X., Cheng Q., Zhang G.F. (2025). Elevated propionate and its association with neurological dysfunctions in propionic acidemia. Front. Mol. Neurosci..

[106] Hao C., Gao Z., Liu X., Rong Z., Jia J., Kang K., Guo W., Li J. (2020). Intravenous administration of sodium propionate induces antidepressant or prodepressant effect in a dose dependent manner. Sci. Rep..

[107] Steliou K., Boosalis M.S., Perrine S.P., Sangerman J., Faller D.V. (2012). Butyrate histone deacetylase inhibitors. Biores Open Access.

[108] Chakraborty P., Laird A.S. (2025). Understanding activity of butyrate at a cellular level. Neural Regen. Res..

[109] Stilling R.M., van der Wouw M., Clarke G., Stanton C., Dinan T.G., Cryan J.F. (2016). The neuropharmacology of butyrate: The bread and butter of the microbiota-gut-brain axis?. Neurochem. Int..

[110] Miletta M.C., Petkovic V., Eble A., Ammann R.A., Fluck C.E., Mullis P.E. (2014). Butyrate increases intracellular calcium levels and enhances growth hormone release from rat anterior pituitary cells via the G-protein-coupled receptors GPR41 and 43. PLoS ONE.

[111] Bourassa M.W., Alim I., Bultman S.J., Ratan R.R. (2016). Butyrate, neuroepigenetics and the gut microbiome: Can a high fiber diet improve brain health?. Neurosci. Lett..

[112] Kalkan A.E., BinMowyna M.N., Raposo A., Ahmad M.F., Ahmed F., Otayf A.Y., Carrascosa C., Saraiva A., Karav S. (2025). Beyond the Gut: Unveiling Butyrate’s Global Health Impact Through Gut Health and Dysbiosis-Related Conditions: A Narrative Review. Nutrients.

[113] Duan W.X., Xie W.Y., Ying C., Fen W., Cheng X.Y., Mao C.J., Liu J.Y., Liu C.F. (2025). Butyrate improves abnormal sleep architecture in a Parkinson’s disease mouse model via BDNF/TrkB signaling. npj Park. Dis..

[114] Garcia-Serrano A.M., Skoug C., Axling U., Korhonen E.R., Teixeira C., Ahren I.L., Mukhopadhya I., Boteva N., Martin J., Scott K. (2024). Butyrate-producing bacteria as probiotic supplement: Beneficial effects on metabolism and modulation of behaviour in an obesity mouse model. Benef. Microbes.

[115] Sampson T.R., Mazmanian S.K. (2015). Control of brain development, function, and behavior by the microbiome. Cell Host Microbe.

[116] Petrut S.M., Bragaru A.M., Munteanu A.E., Moldovan A.D., Moldovan C.A., Rusu E. (2025). Gut over Mind: Exploring the Powerful Gut-Brain Axis. Nutrients.

[117] Ma L., Wang H.B., Hashimoto K. (2025). The vagus nerve: An old but new player in brain-body communication. Brain Behav. Immun..

[118] Oakes P.C., Fisahn C., Iwanaga J., DiLorenzo D., Oskouian R.J., Tubbs R.S. (2016). A history of the autonomic nervous system: Part I: From Galen to Bichat. Childs Nerv. Syst..

[119] Caballero-Villarraso J., Pons-Villarta S., Cruces-Parraga J., Navarrete-Perez A., Camargo A., Moreno J.A., Tunez I., Aguera-Morales E. (2025). Molecular Mechanisms of the Microbiota-Gut-Brain Axis in the Onset and Progression of Stroke. Int. J. Mol. Sci..

[120] Bektas A., Erdal H., Ulusoy M., Uzbay I.T. (2020). Does Serotonin in the intestines make you happy?. Turk. J. Gastroenterol..

[121] Chen Y., Xu J., Chen Y. (2021). Regulation of Neurotransmitters by the Gut Microbiota and Effects on Cognition in Neurological Disorders. Nutrients.

[122] Shashoua V.E., Meyer E.M., Simpkins J.W., Yamamoto J., Crews F.T. (1992). Procedures for Penetrating the Blood-Brain-Barrier: Studies of γ-Aminobutyric Acid (GABA). Treatment of Dementias. Advances in Behavioral Biology.

[123] Niazi S.K. (2023). Non-Invasive Drug Delivery across the Blood-Brain Barrier: A Prospective Analysis. Pharmaceutics.

[124] Spencer N.J., Kyloh M.A., Travis L., Hibberd T.J. (2024). Mechanisms underlying the gut-brain communication: How enterochromaffin (EC) cells activate vagal afferent nerve endings in the small intestine. J. Comp. Neurol..

[125] Olsen L.K., Solis E., McIntire L.K., Hatcher-Solis C.N. (2023). Vagus nerve stimulation: Mechanisms and factors involved in memory enhancement. Front. Hum. Neurosci..

[126] Bremner J.D., Gurel N.Z., Wittbrodt M.T., Shandhi M.H., Rapaport M.H., Nye J.A., Pearce B.D., Vaccarino V., Shah A.J., Park J. (2020). Application of Noninvasive Vagal Nerve Stimulation to Stress-Related Psychiatric Disorders. J. Pers. Med..

[127] Banskota S., Khan W.I. (2022). Gut-derived serotonin and its emerging roles in immune function, inflammation, metabolism and the gut-brain axis. Curr. Opin. Endocrinol. Diabetes Obes..

[128] Yano J.M., Yu K., Donaldson G.P., Shastri G.G., Ann P., Ma L., Nagler C.R., Ismagilov R.F., Mazmanian S.K., Hsiao E.Y. (2015). Indigenous bacteria from the gut microbiota regulate host serotonin biosynthesis. Cell.

[129] Wei L., Singh R., Ghoshal U.C. (2022). Enterochromaffin Cells-Gut Microbiota Crosstalk: Underpinning the Symptoms, Pathogenesis, and Pharmacotherapy in Disorders of Gut-Brain Interaction. J. Neurogastroenterol. Motil..

[130] Nogal A., Valdes A.M., Menni C. (2021). The role of short-chain fatty acids in the interplay between gut microbiota and diet in cardio-metabolic health. Gut Microbes.

[131] Williams B.B., Van Benschoten A.H., Cimermancic P., Donia M.S., Zimmermann M., Taketani M., Ishihara A., Kashyap P.C., Fraser J.S., Fischbach M.A. (2014). Discovery and characterization of gut microbiota decarboxylases that can produce the neurotransmitter tryptamine. Cell Host Microbe.

[132] Zhao W., Wang H., Zheng M., Ni Y. (2025). Bile salt hydrolase: A key player in gut microbiota and its implications for metabolic dysfunction-associated steatotic liver disease. Microbiome Res. Rep..

[133] Gershon M.D., Liu M.T. (2007). Serotonin and neuroprotection in functional bowel disorders. Neurogastroenterol. Motil..

[134] Tao E., Zhu Z., Hu C., Long G., Chen B., Guo R., Fang M., Jiang M. (2022). Potential Roles of Enterochromaffin Cells in Early Life Stress-Induced Irritable Bowel Syndrome. Front. Cell Neurosci..

[135] Wu X., Li J.Y., Lee A., Lu Y.X., Zhou S.Y., Owyang C. (2020). Satiety induced by bile acids is mediated via vagal afferent pathways. JCI Insight.

[136] Ataei P., Kalantari H., Bodnar T.S., Turner R.J. (2025). The gut-brain connection: Microbes’ influence on mental health and psychological disorders. Front. Microbiomes.

[137] Hwang Y.K., Oh J.S. (2025). Interaction of the Vagus Nerve and Serotonin in the Gut-Brain Axis. Int. J. Mol. Sci..

[138] Clapp M., Aurora N., Herrera L., Bhatia M., Wilen E., Wakefield S. (2017). Gut microbiota’s effect on mental health: The gut-brain axis. Clin. Pract..

[139] Safadi J.M., Quinton A.M.G., Lennox B.R., Burnet P.W.J., Minichino A. (2022). Gut dysbiosis in severe mental illness and chronic fatigue: A novel trans-diagnostic construct? A systematic review and meta-analysis. Mol. Psychiatry.

[140] Rathore K., Shukla N., Naik S., Sambhav K., Dange K., Bhuyan D., Imranul Haq Q.M. (2025). The Bidirectional Relationship Between the Gut Microbiome and Mental Health: A Comprehensive Review. Cureus.

[141] Ruohan Z., Ruting W., Hongxi W., Zhenjin H., Jiale L., Rongxin Z., Feng J., Yuanbo S. (2025). Gut microbiota as a novel target for treating anxiety and depression: From mechanisms to multimodal interventions. Front. Microbiol..

[142] Yadav M.K., Kumari I., Singh B., Sharma K.K., Tiwari S.K. (2022). Probiotics, prebiotics and synbiotics: Safe options for next-generation therapeutics. Appl. Microbiol. Biotechnol..

[143] Dinan T.G., Stanton C., Cryan J.F. (2013). Psychobiotics: A novel class of psychotropic. Biol. Psychiatry.

[144] Sliwka A., Polak-Berecka M., Zdybel K., Zelek-Molik A., Wasko A. (2025). Psychobiotics in Depression: Sources, Metabolites, and Treatment-A Systematic Review. Nutrients.

[145] Zhu R., Fang Y., Li H., Liu Y., Wei J., Zhang S., Wang L., Fan R., Wang L., Li S. (2023). Psychobiotic Lactobacillus plantarum JYLP-326 relieves anxiety, depression, and insomnia symptoms in test anxious college via modulating the gut microbiota and its metabolism. Front. Immunol..

[146] Marin I.A., Goertz J.E., Ren T., Rich S.S., Onengut-Gumuscu S., Farber E., Wu M., Overall C.C., Kipnis J., Gaultier A. (2017). Microbiota alteration is associated with the development of stress-induced despair behavior. Sci. Rep..

[147] Wang S., Ishima T., Zhang J., Qu Y., Chang L., Pu Y., Fujita Y., Tan Y., Wang X., Hashimoto K. (2020). Ingestion of Lactobacillus intestinalis and Lactobacillus reuteri causes depression- and anhedonia-like phenotypes in antibiotic-treated mice via the vagus nerve. J. Neuroinflammation.

[148] Oroojzadeh P., Bostanabad S.Y., Lotfi H. (2022). Psychobiotics: The Influence of Gut Microbiota on the Gut-Brain Axis in Neurological Disorders. J. Mol. Neurosci..

[149] Kojima M., Hosoda H., Date Y., Nakazato M., Matsuo H., Kangawa K. (1999). Ghrelin is a growth-hormone-releasing acylated peptide from stomach. Nature.

[150] Yang J., Brown M.S., Liang G., Grishin N.V., Goldstein J.L. (2008). Identification of the acyltransferase that octanoylates ghrelin, an appetite-stimulating peptide hormone. Cell.

[151] Rhea E.M., Salameh T.S., Gray S., Niu J., Banks W.A., Tong J. (2018). Ghrelin transport across the blood-brain barrier can occur independently of the growth hormone secretagogue receptor. Mol. Metab..

[152] Fry M., Ferguson A.V. (2010). Ghrelin: Central nervous system sites of action in regulation of energy balance. Int. J. Pept..

[153] Date Y. (2012). Ghrelin and the vagus nerve. Methods Enzymol..

[154] Howick K., Griffin B.T., Cryan J.F., Schellekens H. (2017). From Belly to Brain: Targeting the Ghrelin Receptor in Appetite and Food Intake Regulation. Int. J. Mol. Sci..

[155] Strandwitz P. (2018). Neurotransmitter modulation by the gut microbiota. Brain Res..

[156] Kakee A., Takanaga H., Terasaki T., Naito M., Tsuruo T., Sugiyama Y. (2001). Efflux of a suppressive neurotransmitter, GABA, across the blood-brain barrier. J. Neurochem..

[157] Takanaga H., Ohtsuki S., Hosoya K., Terasaki T. (2001). GAT2/BGT-1 as a system responsible for the transport of gamma-aminobutyric acid at the mouse blood-brain barrier. J. Cereb. Blood Flow Metab..

[158] Loscher W., Potschka H. (2005). Blood-brain barrier active efflux transporters: ATP-binding cassette gene family. NeuroRx.

[159] Ohtsuki S. (2004). New aspects of the blood-brain barrier transporters; its physiological roles in the central nervous system. Biol. Pharm. Bull..

[160] Terasaki T., Ohtsuki S. (2005). Brain-to-blood transporters for endogenous substrates and xenobiotics at the blood-brain barrier: An overview of biology and methodology. NeuroRx.

[161] Druszczynska M., Sadowska B., Kulesza J., Gasienica-Gliwa N., Kulesza E., Fol M. (2024). The Intriguing Connection Between the Gut and Lung Microbiomes. Pathogens.

[162] Miquel S., Martin R., Bridonneau C., Robert V., Sokol H., Bermudez-Humaran L.G., Thomas M., Langella P. (2014). Ecology and metabolism of the beneficial intestinal commensal bacterium Faecalibacterium prausnitzii. Gut Microbes.

[163] Trompette A., Gollwitzer E.S., Yadava K., Sichelstiel A.K., Sprenger N., Ngom-Bru C., Blanchard C., Junt T., Nicod L.P., Harris N.L. (2014). Gut microbiota metabolism of dietary fiber influences allergic airway disease and hematopoiesis. Nat. Med..

[164] Roager H.M., Licht T.R. (2018). Microbial tryptophan catabolites in health and disease. Nat. Commun..

[165] Hsu C.L., Schnabl B. (2023). The gut-liver axis and gut microbiota in health and liver disease. Nat. Rev. Microbiol..

[166] Pant K., Venugopal S.K., Lorenzo Pisarello M.J., Gradilone S.A. (2023). The Role of Gut Microbiome-Derived Short-Chain Fatty Acid Butyrate in Hepatobiliary Diseases. Am. J. Pathol..

[167] Cui C., Gao S., Shi J., Wang K. (2025). Gut-Liver Axis: The Role of Intestinal Microbiota and Their Metabolites in the Progression of Metabolic Dysfunction-Associated Steatotic Liver Disease. Gut Liver.

[168] Wang J., Zhao Q., Zhang S., Liu J., Fan X., Han B., Hou Y., Ai X. (2026). Microbial short chain fatty acids: Effective histone deacetylase inhibitors in immune regulation (Review). Int. J. Mol. Med..

[169] Licciardi P.V., Ververis K., Karagiannis T.C. (2011). Histone deacetylase inhibition and dietary short-chain Fatty acids. ISRN Allergy.

[170] Kopczynska J., Kowalczyk M. (2024). The potential of short-chain fatty acid epigenetic regulation in chronic low-grade inflammation and obesity. Front. Immunol..

[171] Munte E., Hartmann P. (2025). The Role of Short-Chain Fatty Acids in Metabolic Dysfunction-Associated Steatotic Liver Disease and Other Metabolic Diseases. Biomolecules.

[172] Hollenbeck C.B. (2012). An introduction to the nutrition and metabolism of choline. Cent. Nerv. Syst. Agents Med. Chem..

[173] James K.L., Gertz E.R., Kirschke C.P., Allayee H., Huang L., Kable M.E., Newman J.W., Stephensen C.B., Bennett B.J. (2023). Trimethylamine N-Oxide Response to a Mixed Macronutrient Tolerance Test in a Cohort of Healthy United States Adults. Int. J. Mol. Sci..

[174] Tan X., Liu Y., Long J., Chen S., Liao G., Wu S., Li C., Wang L., Ling W., Zhu H. (2019). Trimethylamine N-Oxide Aggravates Liver Steatosis through Modulation of Bile Acid Metabolism and Inhibition of Farnesoid X Receptor Signaling in Nonalcoholic Fatty Liver Disease. Mol. Nutr. Food Res..

[175] Leon-Mimila P., Villamil-Ramirez H., Li X.S., Shih D.M., Hui S.T., Ocampo-Medina E., Lopez-Contreras B., Moran-Ramos S., Olivares-Arevalo M., Grandini-Rosales P. (2021). Trimethylamine N-oxide levels are associated with NASH in obese subjects with type 2 diabetes. Diabetes Metab..

[176] Gao Q., Li T., Al-Ansi W., Fan M., Qian H., Li Y., Wang L. (2025). TMAO Accumulation Induced by Hyperglycemia Triggers Endoplasmic Reticulum Stress in the Mouse Liver. Mol. Nutr. Food Res..

[177] Xia L., Wang Z., Chen X. (2025). TMAO Induces Vascular Endothelial Cells Pyroptosis Through TET2-CYTB-ROS Pathway. J. Inflamm. Res..

[178] Guo J., Wang Y., Zhang D., Shi C., Zhang X., Li X., Gong Z. (2025). The FMO3/TMAO/HSP90beta axis aggravates MAFLD by disrupting mitochondrial protein homeostasis. Cell Signal.

[179] Kruglov V., In Hwa J., Camell C.D. (2024). Inflammaging and fatty acid oxidation in monocytes and macrophages. Immunometabolism.

[180] Wang B., Zhou Z., Li L. (2022). Gut Microbiota Regulation of AHR Signaling in Liver Disease. Biomolecules.

[181] Jin U.H., Lee S.O., Sridharan G., Lee K., Davidson L.A., Jayaraman A., Chapkin R.S., Alaniz R., Safe S. (2014). Microbiome-derived tryptophan metabolites and their aryl hydrocarbon receptor-dependent agonist and antagonist activities. Mol. Pharmacol..

[182] Gryp T., Vanholder R., Vaneechoutte M., Glorieux G. (2017). P-Cresyl Sulfate. Toxins.

[183] Saito Y., Sato T., Nomoto K., Tsuji H. (2018). Identification of phenol- and p-cresol-producing intestinal bacteria by using media supplemented with tyrosine and its metabolites. FEMS Microbiol. Ecol..

[184] Renaldi R., Wiguna T., Persico A.M., Tanra A.J. (2025). P-Cresol and p-Cresyl Sulphate Boost Oxidative Stress: A Systematic Review of Recent Evidence. Basic Clin. Pharmacol. Toxicol..

[185] Watanabe H., Miyamoto Y., Enoki Y., Ishima Y., Kadowaki D., Kotani S., Nakajima M., Tanaka M., Matsushita K., Mori Y. (2015). P-Cresyl sulfate, a uremic toxin, causes vascular endothelial and smooth muscle cell damages by inducing oxidative stress. Pharmacol. Res. Perspect..

[186] Watanabe H., Miyamoto Y., Honda D., Tanaka H., Wu Q., Endo M., Noguchi T., Kadowaki D., Ishima Y., Kotani S. (2013). P-Cresyl sulfate causes renal tubular cell damage by inducing oxidative stress by activation of NADPH oxidase. Kidney Int..

[187] Ahmed S., Sparidans R.W., Vernooij R.W.M., Mihaila S.M., Broekhuizen R., Goldschmeding R., Nguyen T.Q., Masereeuw R., Gerritsen K.G.F. (2025). Protein-bound uremic toxin clearance as biomarker of kidney tubular function in diabetic kidney disease. Sci. Rep..

[188] Tanaka H., Iwasaki Y., Yamato H., Mori Y., Komaba H., Watanabe H., Maruyama T., Fukagawa M. (2013). P-Cresyl sulfate induces osteoblast dysfunction through activating JNK and p38 MAPK pathways. Bone.

[189] Sun C.Y., Li J.R., Wang Y.Y., Lin S.Y., Ou Y.C., Lin C.J., Wang J.D., Liao S.L., Chen C.J. (2020). P-Cresol Sulfate Caused Behavior Disorders and Neurodegeneration in Mice with Unilateral Nephrectomy Involving Oxidative Stress and Neuroinflammation. Int. J. Mol. Sci..

[190] Flynn C.K., Adams J.B., Krajmalnik-Brown R., Khoruts A., Sadowsky M.J., Nirmalkar K., Takyi E., Whiteley P. (2025). Review of Elevated Para-Cresol in Autism and Possible Impact on Symptoms. Int. J. Mol. Sci..

[191] Corte-Iglesias V., Saiz M.L., Andrade-Lopez A.C., Salazar N., Bernet C.R., Martin-Martin C., Borra J.M., Lozano J.J., Aransay A.M., Diaz-Corte C. (2024). Propionate and butyrate counteract renal damage and progression to chronic kidney disease. Nephrol. Dial. Transpl..

[192] Li Y.J., Ma J., Loh Y.W., Chadban S.J., Wu H. (2023). Short-chain fatty acids directly exert anti-inflammatory responses in podocytes and tubular epithelial cells exposed to high glucose. Front. Cell Dev. Biol..

[193] Wang S., Lv D., Jiang S., Jiang J., Liang M., Hou F., Chen Y. (2019). Quantitative reduction in short-chain fatty acids, especially butyrate, contributes to the progression of chronic kidney disease. Clin. Sci..

[194] Tian X., Sun L., Guo S., Yuan L., Zhang T., Huang C., He T., Jiang Q., Zeng Y. (2025). Gut microbiota-derived SCFAs and MetS-related nephropathy. Front. Nutr..

[195] Li L., Ma L., Fu P. (2017). Gut microbiota-derived short-chain fatty acids and kidney diseases. Drug Des. Dev. Ther..

[196] Liu P., Jin M., Hu P., Sun W., Tang Y., Wu J., Zhang D., Yang L., He H., Xu X. (2024). Gut microbiota and bile acids: Metabolic interactions and impacts on diabetic kidney disease. Curr. Res. Microb. Sci..

[197] Guo Y., Luo T., Xie G., Zhang X. (2023). Bile acid receptors and renal regulation of water homeostasis. Front. Physiol..

[198] Yang X., Delsante M., Daneshpajouhnejad P., Fenaroli P., Mandell K.P., Wang X., Takahashi S., Halushka M.K., Kopp J.B., Levi M. (2024). Bile Acid Receptor Agonist Reverses Transforming Growth Factor-beta1-Mediated Fibrogenesis in Human Induced Pluripotent Stem Cells-Derived Kidney Organoids. Lab. Investig..

[199] Pavlova E.K., Samartsev V.N., Pavlova S.I., Dubinin M.V. (2025). Interaction of bile acids with functionally active liver mitochondria: Uncoupling activity, detergent effect, and antioxidant action. J. Bioenerg. Biomembr..

[200] Madella A.M., Van Bergenhenegouwen J., Garssen J., Masereeuw R., Overbeek S.A. (2022). Microbial-Derived Tryptophan Catabolites, Kidney Disease and Gut Inflammation. Toxins.

[201] Tennoune N., Andriamihaja M., Blachier F. (2022). Production of Indole and Indole-Related Compounds by the Intestinal Microbiota and Consequences for the Host: The Good, the Bad, and the Ugly. Microorganisms.

[202] Banoglu E., Jha G.G., King R.S. (2001). Hepatic microsomal metabolism of indole to indoxyl, a precursor of indoxyl sulfate. Eur. J. Drug Metab. Pharmacokinet..

[203] Banoglu E., King R.S. (2002). Sulfation of indoxyl by human and rat aryl (phenol) sulfotransferases to form indoxyl sulfate. Eur. J. Drug Metab. Pharmacokinet..

[204] Bolati D., Shimizu H., Yisireyili M., Nishijima F., Niwa T. (2013). Indoxyl sulfate, a uremic toxin, downregulates renal expression of Nrf2 through activation of NF-kappaB. BMC Nephrol..

[205] Ribeiro A., Liu F., Srebrzynski M., Rother S., Adamowicz K., Wadowska M., Steiger S., Anders H.J., Schmaderer C., Koziel J. (2023). Uremic Toxin Indoxyl Sulfate Promotes Macrophage-Associated Low-Grade Inflammation and Epithelial Cell Senescence. Int. J. Mol. Sci..

[206] Jha P.K., Nakano T., Itto L.Y.U., Barbeiro M.C., Lupieri A., Aikawa E., Aikawa M. (2025). Vascular inflammation in chronic kidney disease: The role of uremic toxins in macrophage activation. Front. Cardiovasc. Med..

[207] Nakano T., Watanabe H., Imafuku T., Tokumaru K., Fujita I., Arimura N., Maeda H., Tanaka M., Matsushita K., Fukagawa M. (2021). Indoxyl Sulfate Contributes to mTORC1-Induced Renal Fibrosis via The OAT/NADPH Oxidase/ROS Pathway. Toxins.

[208] Li Y., Yan J., Wang M., Lv J., Yan F., Chen J. (2021). Uremic toxin indoxyl sulfate promotes proinflammatory macrophage activation by regulation of beta-catenin and YAP pathways. J. Mol. Histol..

[209] Cheng T.H., Ma M.C., Liao M.T., Zheng C.M., Lu K.C., Liao C.H., Hou Y.C., Liu W.C., Lu C.L. (2020). Indoxyl Sulfate, a Tubular Toxin, Contributes to the Development of Chronic Kidney Disease. Toxins.

[210] Alobaidi S. (2025). The gut-kidney axis in chronic kidney disease: Mechanisms, microbial metabolites, and microbiome-targeted therapeutics. Front. Med..

[211] Austin H.A., Goldin H., Gaydos D., Preuss H.G. (1983). Polyamine metabolism in compensatory renal growth. Kidney Int..

[212] Zahedi K., Barone S., Soleimani M. (2022). Polyamines and Their Metabolism: From the Maintenance of Physiological Homeostasis to the Mediation of Disease. Med. Sci..

[213] Arthur R., Jamwal S., Kumar P. (2024). A review on polyamines as promising next-generation neuroprotective and anti-aging therapy. Eur. J. Pharmacol..

[214] Zahedi K., Barone S., Brooks M., Stewart T.M., Foley J.R., Nwafor A., Casero R.A., Soleimani M. (2024). Polyamine Catabolism and Its Role in Renal Injury and Fibrosis in Mice Subjected to Repeated Low-Dose Cisplatin Treatment. Biomedicines.

[215] Mafe A.N., Busselberg D. (2026). The Diet-Microbiota-Polyamine Axis in Intestinal Aging: Microbial Pathways, Functional Foods, and Physiological Implications. Nutrients.

[216] Kitada Y., Muramatsu K., Toju H., Kibe R., Benno Y., Kurihara S., Matsumoto M. (2018). Bioactive polyamine production by a novel hybrid system comprising multiple indigenous gut bacterial strategies. Sci. Adv..

[217] Tofalo R., Cocchi S., Suzzi G. (2019). Polyamines and Gut Microbiota. Front. Nutr..

[218] Sugiyama Y., Nara M., Sakanaka M., Kitakata A., Okuda S., Kurihara S. (2018). Analysis of polyamine biosynthetic- and transport ability of human indigenous Bifidobacterium. Biosci. Biotechnol. Biochem..

[219] Hobley L., Li B., Wood J.L., Kim S.H., Naidoo J., Ferreira A.S., Khomutov M., Khomutov A., Stanley-Wall N.R., Michael A.J. (2017). Spermidine promotes Bacillus subtilis biofilm formation by activating expression of the matrix regulator slrR. J. Biol. Chem..

[220] Kim G., Kim M., Kim M., Park C., Yoon Y., Lim D.H., Yeo H., Kang S., Lee Y.G., Beak N.I. (2021). Spermidine-induced recovery of human dermal structure and barrier function by skin microbiome. Commun. Biol..

[221] Drzewiecka D. (2016). Significance and Roles of *Proteus* spp. Bacteria in Natural Environments. Microb. Ecol..

[222] Kim J.Y., Dao H. (2026). Physiology, Integument. StatPearls.

[223] Salem I., Ramser A., Isham N., Ghannoum M.A. (2018). The Gut Microbiome as a Major Regulator of the Gut-Skin Axis. Front. Microbiol..

[224] Lee Y.B., Byun E.J., Kim H.S. (2019). Potential Role of the Microbiome in Acne: A Comprehensive Review. J. Clin. Med..

[225] Thye A.Y., Bah Y.R., Law J.W., Tan L.T., He Y.W., Wong S.H., Thurairajasingam S., Chan K.G., Lee L.H., Letchumanan V. (2022). Gut-Skin Axis: Unravelling the Connection between the Gut Microbiome and Psoriasis. Biomedicines.

[226] Hou B., Shao H., Yuan D., Tham E.H. (2025). Skin and gut microbiome in atopic dermatitis: Mechanisms and therapeutic opportunities. Pediatr. Allergy Immunol..

[227] Qu B., Zhang X.E., Feng H., Yan B., Bai Y., Liu S., He Y. (2024). Microbial perspective on the skin-gut axis and atopic dermatitis. Open Life Sci..

[228] Kendall S.N. (2004). Remission of rosacea induced by reduction of gut transit time. Clin. Exp. Dermatol..

[229] Chilicka K., Dziendziora-Urbinska I., Szygula R., Asanova B., Nowicka D. (2022). Microbiome and Probiotics in Acne Vulgaris-A Narrative Review. Life.

[230] Zhao Y., Yu C., Zhang J., Yao Q., Zhu X., Zhou X. (2025). The gut-skin axis: Emerging insights in understanding and treating skin diseases through gut microbiome modulation (Review). Int. J. Mol. Med..

[231] Grice E.A., Segre J.A. (2011). The skin microbiome. Nat. Rev. Microbiol..

[232] Paichitrojjana A. (2022). Demodex: The worst enemies are the ones that used to be friends. Dermatol. Rep..

[233] Rather P.A., Hassan I. (2014). Human demodex mite: The versatile mite of dermatological importance. Indian J. Dermatol..

[234] DeAngelis Y.M., Saunders C.W., Johnstone K.R., Reeder N.L., Coleman C.G., Kaczvinsky J.R., Gale C., Walter R., Mekel M., Lacey M.P. (2007). Isolation and expression of a Malassezia globosa lipase gene, LIP1. J. Investig. Dermatol..

[235] Pyzia J., Mankowska K., Czepita M., Kot K., Lanocha-Arendarczyk N., Czepita D., Kosik-Bogacka D.I. (2023). Demodex Species and Culturable Microorganism Co-Infestations in Patients with Blepharitis. Life.

[236] El-Moamly A., El-Swify O. (2025). Raising awareness of Demodex mites: A neglected cause of skin disease. Infection.

[237] Jarmuda S., O’Reilly N., Zaba R., Jakubowicz O., Szkaradkiewicz A., Kavanagh K. (2012). Potential role of Demodex mites and bacteria in the induction of rosacea. J. Med. Microbiol..

[238] Gulbasaran F., Sarimustafa S., Ozbagcivan O., Kose S., Avci E. (2024). Investigation of Factors Associated with Gut Microbiota in Demodex-associated Skin Conditions. Turk. Parazitol. Derg..

[239] Daou H., Paradiso M., Hennessy K., Seminario-Vidal L. (2021). Rosacea and the Microbiome: A Systematic Review. Dermatol. Ther..

[240] Umbach A.K., Stegelmeier A.A., Neufeld J.D. (2021). Archaea Are Rare and Uncommon Members of the Mammalian Skin Microbiome. mSystems.

[241] Probst A.J., Auerbach A.K., Moissl-Eichinger C. (2013). Archaea on human skin. PLoS ONE.

[242] Smythe P., Wilkinson H.N. (2023). The Skin Microbiome: Current Landscape and Future Opportunities. Int. J. Mol. Sci..

[243] Cogen A.L., Yamasaki K., Muto J., Sanchez K.M., Crotty Alexander L., Tanios J., Lai Y., Kim J.E., Nizet V., Gallo R.L. (2010). Staphylococcus epidermidis antimicrobial delta-toxin (phenol-soluble modulin-gamma) cooperates with host antimicrobial peptides to kill group A Streptococcus. PLoS ONE.

[244] Qi P., Gong F., Leng M., Wei Z. (2025). Beneficial perspective on Staphylococcus epidermidis: A crucial species for skin homeostasis and pathogen defense. Front. Immunol..

[245] Suri H., Suri H., Nagda N., Misra T., Vuppu S. (2025). Current perspectives on the human skin microbiome: Functional insights and strategies for therapeutic modulation. BioMed Pharmacother..

[246] Hong J.Y., Kwon D., Park K.Y. (2025). Microbiome-Based Interventions for Skin Aging and Barrier Function: A Comprehensive Review. Ann. Dermatol..

[247] Lyu Y., Shen J., Che Y., Dai L. (2025). Skin microbiome engineering: Challenges and opportunities in skin diseases treatment. IMetaOmics.

[248] van Smeden J., Al-Khakany H., Wang Y., Visscher D., Stephens N., Absalah S., Overkleeft H.S., Aerts J., Hovnanian A., Bouwstra J.A. (2020). Skin barrier lipid enzyme activity in Netherton patients is associated with protease activity and ceramide abnormalities. J. Lipid Res..

[249] Boer D.E.C., van Smeden J., Al-Khakany H., Melnik E., van Dijk R., Absalah S., Vreeken R.J., Haenen C.C.P., Lavrijsen A.P.M., Overkleeft H.S. (2020). Skin of atopic dermatitis patients shows disturbed beta-glucocerebrosidase and acid sphingomyelinase activity that relates to changes in stratum corneum lipid composition. Biochim. Biophys. Acta Mol. Cell Biol. Lipids.

[250] Zheng Y., Hunt R.L., Villaruz A.E., Fisher E.L., Liu R., Liu Q., Cheung G.Y.C., Li M., Otto M. (2022). Commensal Staphylococcus epidermidis contributes to skin barrier homeostasis by generating protective ceramides. Cell Host Microbe.

[251] Li M., Kopylova E., Mao J., Namkoong J., Sanders J., Wu J. (2024). Microbiome and lipidomic analysis reveal the interplay between skin bacteria and lipids in a cohort study. Front. Microbiol..

[252] Brooks S.G., Mahmoud R.H., Lin R.R., Fluhr J.W., Yosipovitch G. (2025). The Skin Acid Mantle: An Update on Skin pH. J. Investig. Dermatol..

[253] Micu A.E., Popescu I.A., Halip I.A., Mocanu M., Vata D., Hulubencu A.L., Gheuca-Solovastru D.F., Gheuca-Solovastru L. (2025). From Gut Dysbiosis to Skin Inflammation in Atopic Dermatitis: Probiotics and the Gut-Skin Axis-Clinical Outcomes and Microbiome Implications. Int. J. Mol. Sci..

[254] Maestro M.A., Molnar F., Carlberg C. (2019). Vitamin D and Its Synthetic Analogs. J. Med. Chem..

[255] Lee S.Y., Lee E., Park Y.M., Hong S.J. (2018). Microbiome in the Gut-Skin Axis in Atopic Dermatitis. Allergy Asthma Immunol. Res..

[256] Ashkanani A., Ashkanani G., Yousef M., Rob M., Al-Marri M., Naseem N., Laws S., Chaari A. (2025). Microbiome and Skin Health: A Systematic Review of Nutraceutical Interventions, Disease Severity, Inflammation, and Gut Microbiota. Microorganisms.

[257] Harrandah A.M. (2025). The Oral-Gut-Systemic Axis: Emerging Insights into Periodontitis, Microbiota Dysbiosis, and Systemic Disease Interplay. Diagnostics.

[258] Kamer A.R., Pushalkar S., Hamidi B., Janal M.N., Tang V., Annam K.R.C., Palomo L., Gulivindala D., Glodzik L., Saxena D. (2024). Periodontal Inflammation and Dysbiosis Relate to Microbial Changes in the Gut. Microorganisms.

[259] Gan G., Chen R., Zheng P., Long K., Cheng K.K.Y., Sulaiman J.E., Huang X. (2025). Oral pathogens meet the gut microbiome: New mechanistic insights on systemic disease. Front. Cell Infect. Microbiol..

[260] Azzolino D., Carnevale-Schianca M., Santacroce L., Colella M., Felicetti A., Terranova L., Castrejon-Perez R.C., Garcia-Godoy F., Lucchi T., Passarelli P.C. (2025). The Oral-Gut Microbiota Axis Across the Lifespan: New Insights on a Forgotten Interaction. Nutrients.

[261] Yamazaki K., Kamada N. (2024). Exploring the oral-gut linkage: Interrelationship between oral and systemic diseases. Mucosal Immunol..

[262] Tan X., Wang Y., Gong T. (2023). The interplay between oral microbiota, gut microbiota and systematic diseases. J. Oral Microbiol..

[263] Salama R.A.A., Msalat O.F., Fouad M.M., Alhammadi M., Elsheikh S., Nasser R.A. (2026). The Oral Microbiome-Nitrate-Nitrite-Nitric Oxide Axis and Cardiovascular Health: A Narrative Review. J. Clin. Med..

[264] Rosier B.T., Moya-Gonzalvez E.M., Corell-Escuin P., Mira A. (2020). Isolation and Characterization of Nitrate-Reducing Bacteria as Potential Probiotics for Oral and Systemic Health. Front. Microbiol..

[265] Hezel M.P., Weitzberg E. (2015). The oral microbiome and nitric oxide homoeostasis. Oral Dis..

[266] Moran S.P., Rosier B.T., Henriquez F.L., Burleigh M.C. (2024). The effects of nitrate on the oral microbiome: A systematic review investigating prebiotic potential. J. Oral Microbiol..

[267] Gonzalez-Soltero R., Bailen M., de Lucas B., Ramirez-Goercke M.I., Pareja-Galeano H., Larrosa M. (2020). Role of Oral and Gut Microbiota in Dietary Nitrate Metabolism and Its Impact on Sports Performance. Nutrients.

[268] Iijima K., Henry E., Moriya A., Wirz A., Kelman A.W., McColl K.E. (2002). Dietary nitrate generates potentially mutagenic concentrations of nitric oxide at the gastroesophageal junction. Gastroenterology.

[269] Lundberg J.O., Weitzberg E., Gladwin M.T. (2008). The nitrate-nitrite-nitric oxide pathway in physiology and therapeutics. Nat. Rev. Drug Discov..

[270] Sobko T., Huang L., Midtvedt T., Norin E., Gustafsson L.E., Norman M., Jansson E.A., Lundberg J.O. (2006). Generation of NO by probiotic bacteria in the gastrointestinal tract. Free Radic. Biol. Med..

[271] Shi H., Huang L., Zhang J.H., Shen C., Zhang N., Lv C., Shao L., Li M., Sun Z., Shi L. (2026). Gut Microbiota Regulates Brain-Bone Axis to Influence Osteoporosis Pathogenesis and Treatment. Research.

[272] Wang Y., Xu M., Dong Q., Lin X., Zhang Q., Shi H., Lin X., Liu H., Yu M., Shen J. (2026). The gut-bone axis: Impact of diet on gut microbiome and osteoporosis. Bone Res..

[273] Savanelli M.C., Barrea L., Macchia P.E., Savastano S., Falco A., Renzullo A., Scarano E., Nettore I.C., Colao A., Di Somma C. (2017). Preliminary results demonstrating the impact of Mediterranean diet on bone health. J. Transl. Med..

[274] La Reau A.J., Suen G. (2018). The Ruminococci: Key symbionts of the gut ecosystem. J. Microbiol..

[275] Nie K., Ma K., Luo W., Shen Z., Yang Z., Xiao M., Tong T., Yang Y., Wang X. (2021). Roseburia intestinalis: A Beneficial Gut Organism From the Discoveries in Genus and Species. Front. Cell Infect. Microbiol..

[276] Lucas S., Omata Y., Hofmann J., Bottcher M., Iljazovic A., Sarter K., Albrecht O., Schulz O., Krishnacoumar B., Kronke G. (2018). Short-chain fatty acids regulate systemic bone mass and protect from pathological bone loss. Nat. Commun..

[277] Huang S.C., He Y.F., Chen P., Liu K.L., Shaukat A. (2023). Gut microbiota as a target in the bone health of livestock and poultry: Roles of short-chain fatty acids. Anim. Dis..

[278] Grahnemo L., Kambur O., Lahti L., Jousilahti P., Niiranen T., Knight R., Salomaa V., Havulinna A.S., Ohlsson C. (2024). Associations between gut microbiota and incident fractures in the FINRISK cohort. npj Biofilms Microbiomes.

[279] Patterson G.T., Osorio E.Y., Peniche A., Dann S.M., Cordova E., Preidis G.A., Suh J.H., Ito I., Saldarriaga O.A., Loeffelholz M. (2022). Pathologic Inflammation in Malnutrition Is Driven by Proinflammatory Intestinal Microbiota, Large Intestine Barrier Dysfunction, and Translocation of Bacterial Lipopolysaccharide. Front. Immunol..

[280] Subramaniam S., Fletcher C. (2018). Trimethylamine N-oxide: Breathe new life. Br. J. Pharmacol..

[281] Lin H., Liu T., Li X., Gao X., Wu T., Li P. (2020). The role of gut microbiota metabolite trimethylamine N-oxide in functional impairment of bone marrow mesenchymal stem cells in osteoporosis disease. Ann. Transl. Med..

[282] Zhao Y., Wang C., Qiu F., Liu J., Xie Y., Lin Z., He J., Chen J. (2024). Trimethylamine-N-oxide promotes osteoclast differentiation and oxidative stress by activating NF-kappaB pathway. Aging.

[283] Sevcikova A., Martiniakova M., Omelka R., Stevurkova V., Ciernikova S. (2024). The Link Between the Gut Microbiome and Bone Metastasis. Int. J. Mol. Sci..

[284] Guss J.D., Horsfield M.W., Fontenele F.F., Sandoval T.N., Luna M., Apoorva F., Lima S.F., Bicalho R.C., Singh A., Ley R.E. (2017). Alterations to the Gut Microbiome Impair Bone Strength and Tissue Material Properties. J. Bone Min. Res..

[285] Shi R., Yang Z., Zhuang Z. (2026). Mechanisms of the brain-gut-bone axis on bone metabolism: Mediated by serotonin (5-HT) and associated molecules. Sci. Prog..

[286] Snelson M., Muralitharan R.R., Liu C.F., Marko L., Forslund S.K., Marques F.Z., Tang W.H.W. (2025). Gut-Heart Axis: The Role of Gut Microbiota and Metabolites in Heart Failure. Circ. Res..

[287] Kondapalli N., Katari V., Dalal K.K., Paruchuri S., Thodeti C.K. (2025). Microbiota in Gut-Heart Axis: Metabolites and Mechanisms in Cardiovascular Disease. Compr. Physiol..

[288] Franzosa E.A., Sirota-Madi A., Avila-Pacheco J., Fornelos N., Haiser H.J., Reinker S., Vatanen T., Hall A.B., Mallick H., McIver L.J. (2019). Gut microbiome structure and metabolic activity in inflammatory bowel disease. Nat. Microbiol..

[289] Chen B., Collen L.V., Mowat C., Isaacs K.L., Singh S., Kane S.V., Farraye F.A., Snapper S., Jneid H., Lavie C.J. (2022). Inflammatory Bowel Disease and Cardiovascular Diseases. Am. J. Med..

[290] Romano K.A., Nemet I., Prasad Saha P., Haghikia A., Li X.S., Mohan M.L., Lovano B., Castel L., Witkowski M., Buffa J.A. (2023). Gut Microbiota-Generated Phenylacetylglutamine and Heart Failure. Circ. Heart Fail.

[291] Epelde F. (2025). The Role of the Gut Microbiota in Heart Failure: Pathophysiological Insights and Future Perspectives. Medicina.

[292] Yu L., Meng G., Huang B., Zhou X., Stavrakis S., Wang M., Li X., Zhou L., Wang Y., Wang M. (2018). A potential relationship between gut microbes and atrial fibrillation: Trimethylamine N-oxide, a gut microbe-derived metabolite, facilitates the progression of atrial fibrillation. Int. J. Cardiol..

[293] Chen H.C., Tang T.W.H., Pasaribu S.N.N., Wu D.C., Rey F.E., Hsieh P.C.H. (2026). Gut-Heart Axis in Myocardial Repair: Mechanisms, Cross-Organ Networks, and Therapeutic Opportunities. Circ. Res..

[294] Wang S., Zhang L., Zhao J., Bai M., Lin Y., Chu Q., Gong J., Qiu J., Chen Y. (2024). Intestinal monocarboxylate transporter 1 mediates lactate transport in the gut and regulates metabolic homeostasis of mouse in a sex-dimorphic pattern. Life Metab..

[295] Herrmann D.B., Herz R., Frohlich J. (1985). Role of gastrointestinal tract and liver in acetate metabolism in rat and man. Eur. J. Clin. Investig..

[296] Geistlinger K., Schmidt J.D.R., Beitz E. (2023). Human monocarboxylate transporters accept and relay protons via the bound substrate for selectivity and activity at physiological pH. PNAS Nexus.

[297] Zhang M., Wang Y., Bai Y., Dai L., Guo H. (2022). Monocarboxylate Transporter 1 May Benefit Cerebral Ischemia via Facilitating Lactate Transport From Glial Cells to Neurons. Front. Neurol..

[298] Bonen A. (2000). Lactate transporters (MCT proteins) in heart and skeletal muscles. Med. Sci. Sports Exerc..

[299] Kido Y., Tamai I., Okamoto M., Suzuki F., Tsuji A. (2000). Functional clarification of MCT1-mediated transport of monocarboxylic acids at the blood-brain barrier using in vitro cultured cells and in vivo BUI studies. Pharm. Res..

[300] Liu Z., Sneve M., Haroldson T.A., Smith J.P., Drewes L.R. (2016). Regulation of Monocarboxylic Acid Transporter 1 Trafficking by the Canonical Wnt/beta-Catenin Pathway in Rat Brain Endothelial Cells Requires Cross-talk with Notch Signaling. J. Biol. Chem..

[301] Erny D., Hrabe de Angelis A.L., Jaitin D., Wieghofer P., Staszewski O., David E., Keren-Shaul H., Mahlakoiv T., Jakobshagen K., Buch T. (2015). Host microbiota constantly control maturation and function of microglia in the CNS. Nat. Neurosci..

[302] Rothhammer V., Borucki D.M., Tjon E.C., Takenaka M.C., Chao C.C., Ardura-Fabregat A., de Lima K.A., Gutierrez-Vazquez C., Hewson P., Staszewski O. (2018). Microglial control of astrocytes in response to microbial metabolites. Nature.

[303] Giordano L., Ahmed S., van der Made T.K., Masereeuw R., Mihaila S.M. (2025). Gut microbial-derived short chain fatty acids enhance kidney proximal tubule cell secretory function. BioMed Pharmacother..

[304] Foxcroft L. (2011). Calories & Corsets—A History of Dieting Over 2000 Years.

[305] Sender R., Fuchs S., Milo R. (2016). Revised Estimates for the Number of Human and Bacteria Cells in the Body. PLoS Biol..

[306] Nasrallah H.A. It Takes Guts to be Mentally Ill, Part 1: A Brief Overview of the Human Gut Microbiota. https://www.psychiatrist.com/jcp/it-takes-guts-to-be-mentally-ill-part-1-a-brief-overview-of-the-human-gut-microbiota/.

[307] Zhong Y., Lei Y., Jiang S., Chen D., Wang X., Wang K., Liao T., Liao R., Gan M., Niu L. (2025). Advances in understanding the role of gut microbiota in fat deposition and lipid metabolism. J. Anim. Sci. Biotechnol..

[308] Li G., Jin B., Fan Z. (2022). Mechanisms Involved in Gut Microbiota Regulation of Skeletal Muscle. Oxid. Med. Cell Longev..

[309] Beyoglu D., Idle J.R. (2026). Molecular signatures of the gut microbiota that affect longevity. Biochem. Pharmacol..

